# The quantiles of extreme differences matrix for evaluating discriminant validity

**DOI:** 10.1515/em-2025-0006

**Published:** 2025-08-25

**Authors:** Tyler J. VanderWeele, R. Noah Padgett

**Affiliations:** Departments of Epidemiology and Biostatistics, Harvard T.H. Chan School of Public Health, Boston, MA, USA; Department of Epidemiology, Harvard T.H. Chan School of Public Health, Boston, MA, USA

**Keywords:** discriminant validity, facets, factor analysis, psycho-social constructs, quantiles

## Abstract

When data on multiple indicators of underlying psychosocial constructs are collected, they are often intended as closely related assessments of a relatively unified phenomenon, or alternatively as capturing distinct facets of the phenomenon. Establishing distinctions among construct phenomena, assessments, or indicators is sometimes described as establishing discriminant validity. In the philosophical literature, often extreme instances or limit cases, actual or hypothetical, are used to identify settings in which one phenomenon is present and the other is not, to establish distinctions. We put forward an empirical analogue of this philosophical principle applied to distinctions amongst survey item responses. The quantiles of extreme differences matrix characterizes, for each pair of indicators, how large differences are between indicators at relatively extreme quantiles of the distribution of those differences. We discuss potential uses and properties of this matrix and related matrices for identifying relevant distinctions among indicators or facets of underlying construct phenomena.

## Introduction

In the development of scales and assessments of psychosocial phenomena, a series of indicators is typically employed. Subsequent psychometric evaluation uncovers the empirical properties of these indicators and their relation to one another. Various measures, such as coefficient alpha, evaluate the relative consistency of the values of the individual indicators [1]. Statistical techniques, such as factor analysis, are often employed to evaluate the extent to which a single factor suffices to explain the shared variation among the individual indicators or, alternatively, to identify clusters of indicators that are strongly correlated with one another [2], [3], [4], [5]. In the case of scale development, these various metrics and methods are often employed to establish the reasonableness of using a single composite measure, such as an average, to summarize the indicators as an assessment of the underlying phenomenon under study. Such summary measures are often then used in subsequent empirical research in the biomedical and social sciences. Not infrequently, the values of the individual indicators themselves are ignored. This approach can be and has been moderately successful in uncovering evidence for potentially causal relations between different phenomena [5], 6]. However, the use of the composite measures, ignoring the individual indicators may also obscure information that may be of interest either in its own right, or with regard to nuances in distinct causal effects of various facets of the underlying phenomenon under study [6], [7], [8], [9]. The epidemiologic literature on causal inference has been particularly attentive to these questions of well-defined exposures and interventions, and the complexities of interpreting causal effect estimates of composite exposures, or in settings in which multiple versions of the treatment variable are conceivable [6], [10], [11], [12], [13], [14], [15], [16].

Even independent of considering causal effects, drawing distinctions across constructs, assessments, and indicators is also a fundamental aspect of measure development and psychometric evaluation [17], [18], [19], [20] and aspects of this are sometimes described as the process of establishing “discriminant validity”. These distinctions can be relevant and of interest in their own right, and can further assist in the interpreting subsequent empirical literature.

In contrast to what is common practice within the social and biomedical sciences, which often focuses on averages, both across individuals, and across indicators, it is more typical in philosophical discussions of various phenomena to consider extreme instances or limit cases to establish distinctions. When two closely related concepts, phenomena, or facets of a phenomenon are being considered, philosophers will sometimes consider whether hypothetical cases can be constructed in which, for example, one facet is present, but the other closely related facet is absent. Such hypothetical cases may only rarely arise in practice, and in a given population there may, in actual fact, be no such instances of these cases. Nevertheless, that such hypothetical cases can be constructed is viewed as sufficient to indicate that the facets or phenomena under consideration are distinct. Such distinctions are viewed as important in bringing conceptual clarity, and in understanding more precisely different concepts under consideration. Indeed the principal method of philosophy is, by some, considered simply to be that of “making distinctions” [21].

In this paper, we will propose an empirical analogue, to be employed in conjunction with data collection, to the philosophical approach of considering extreme instances or limit cases. The characterization of the approach is in fact relatively simple. In the context of sample data collection on a series of indicators intended to assess some psychosocial construct, one can consider the difference in individual responses in each pair of indicators. One can then examine the individuals for whom such a difference is particularly extreme, e.g. one can look for, relatively speaking, very large negative, or very large positive, differences. If there are a sufficient number of individuals for whom this difference is particularly extreme, then this might in some cases be considered compelling evidence that the indicators are assessing distinct facets of the underlying psychosocial phenomenon under study. This would arguably still be the case even if the indicators themselves were very strongly positively correlated. It may be that most of the time the two indicators, and the facets of the phenomenon that they assess, are strongly related, but that in at least some instances, i.e. for some individuals, one facet might be strongly present without the other, or vice versa. Examining these extreme differences in the indicators may give insight into such distinctions that are not readily apparent from a more traditional correlation matrix.

Unlike the analogous hypothetical exercise in philosophy, however, when it comes to empirical assessment, it might be thought that a single instance of an extreme difference may not be sufficient to definitively draw an important distinction. This is because, in the context of data collection, it is possible a respondent might have misinterpreted the item or question corresponding to one of the indicators, or that there might have been an error in entry or transcription, or that the respondent was neglectful, or that the respondent intentionally did not answer accurately. The responses of a single participant might then not be viewed as sufficiently reliable to be able to draw firm conclusions about distinctions across indicators. For this reason, it may be more reasonable to examine relatively extreme quantiles of the differences in indicators. If, for example, the 2.5th and 97.5th quantiles in the differences between a pair of indicators across individuals in a sample were examined and found to be relatively extreme, this might be viewed as more reliable evidence of an actual distinction between what these indicators were assessing. Once again, this could in principle occur even if the correlation between the two indicators were fairly high. To more systematically evaluate such evidence we will thus propose reporting such quantiles for all pairs of indicators, or summaries of indicators supposedly corresponding to facets. We will refer to a matrix that reports outer quantiles concerning these relatively extreme differences as the Quantiles of Extreme Differences matrix, which we will also refer to, as a shorthand, as the QED matrix. We will offer some discussion as to when it may, or may not, be reasonable to report such matrices, and also how variations on the proposed QED matrix may also offer insight.

Our proposal to routinely report such QED matrices is not intended as an alternative to a more traditional correlation matrix, or to methods such as factor analysis that utilize them. Rather, we see the QED matrix as a complement to more traditional psychometric evaluation methods. While correlation matrices allow us to see average tendencies in how indicators relate to one another, QED matrices will also allow us to see extreme differences between indicators that may arise for much smaller subsets of individuals. Such distinctions will allow us to see distinctions between indicators that may be present even if the indicators themselves are, for the population, very strongly correlated. As noted above, such distinctions may also help uncover instances in which it may be reasonable to further investigate whether different facets of an underlying construct phenomenon may have different causal relations with various outcomes [6], 7], 9].

## The quantiles of extreme differences matrix: definitions and interpretation

Suppose that in the assessment of some underlying construct, measurements are taken on a series of indicators ( $\left.X_{1},\ldots ,{\text{X}}_{\text{d}}\right)$ . For a given individual the difference between two indicators, *i* and *j*, is given by *D*_*ij*_=*X*_*i*_ − *X*_*j*_. Let $D_{ij}^{\left(q\right)}$ denote the *q*th quantile across individuals of the differences *D*_*ij*_. The Quantiles of Extreme Differences (QED) matrix relative to quantile *q*, which we will denote by $M_{\text{QED}}^{q}$ , is then defined as the matrix for which the *i-j* entry is given by the bivariate pair of quantities $\left(D_{ij}^{\left(q\right)},D_{ij}^{\left(1-q\right)}\right)$ . In other words, the *i-j* entry for the QED matrix is just the *q*th quantile and the (1-*q*)^th^ of the differences between indicator *i* and indicator *j*. The *q*th quantile corresponds to this difference in indicator values for the more extreme individuals for whom indicator *i* is, relatively speaking, small when compared to indicator *j*; the (1-*q*)^th^ quantile of the differences between indicator *i* and indicator *j* corresponds to this difference for the more extreme individuals for whom indicator *i* is, relatively speaking, large when compared to indicator *j*. If either of these outer quantiles, corresponding to extreme differences, is thought to be substantial then this might, in certain circumstances, be taken as compelling evidence that the indicators themselves are evaluating distinct facets of the construct being studied. However, it is important to understand in what contexts, and under what circumstances, it is reasonable to use the QED matrix in this manner. Several interrelated interpretative comments and caveats thus merit attention. We will make these remarks with regard to potential distinctions between specific indicators, but in cases in which specific groups of indicators are thought *a priori* to correspond to distinct facets, the same approaches and comments could be applied to the means of the distinct groups of indicators, a point to which we will return below.

First, the QED matrix is of course relative to the quantile *q*. Smaller values of the quantile *q* correspond to more extreme differences being considered, but of course then also correspond to smaller proportions of the sample under study. It was noted above that it may be preferable to consider the relatively extreme quantiles of the differences *X*_*i*_ − *X*_*j*_, rather than their maximum and minimum values, because the maximum and minimal values would correspond to the responses of a single individual. If that individual misinterpreted the item or question corresponding to one of the indicators, or intentionally misreported, or was neglectful, or there was an error in entry or transcription, then an extreme difference would not constitute evidence for a distinction in what the indicators were assessing. Using extreme quantiles, rather than maximum and minimum values, partially mitigates this concern. However, it does so only to the extent that it is the case that the *q*% reporting the most extreme differences themselves are not misinterpreting or misreporting. Whether this might be so, and whether it is reasonable to think this might be so, will of course depend on the nature of the sample under study, the questions being asked, and also on the size of the sample. If there is reason to doubt the accuracy of the responses, then a larger value of *q* may be desirable.

We would propose, as a default, using a value of *q*=2.5. This would then correspond to examining the 2.5th and 97.5th quantiles of the differences *X*_*i*_ − *X*_*j*_ for each pair of indicators. In a sample of 400 participants, it would require that the 10 participants with the smallest or largest differences in the indicators have unreliable responses on one or both of the indicators for the supposed evidence to be inapplicable. However, again, the choice of *q* may vary by context. With a smaller sample size, or again with a sample in which the accuracy of reporting were of greater concern, a value of *q* greater than 2.5 may be desirable. The larger the value of *q*, the smaller the extreme differences will be in absolute magnitude and when these absolute differences are sufficiently small, the case for a distinction across indicators may be weak. However, a larger value of *q* also indicates that a larger proportion of the respondents would have had to have answered inaccurately for the supposed evidence to be inapplicable, so there are trade-offs in the selection of *q* which need to be evaluated in any given context. In principle, the QED matrices could of course also be presented with more than one value of *q*. In principle it would also be possible to select different values for the upper and lower quantiles. If there were compelling reasons to think over-reporting or under-reporting of certain indicators were more likely one could in principle provide a generalized QED matrix, $M_{\mathrm{QED}}^{q_{1},q_{2}}$ , where each entry were given by $\left(D_{ij}^{\left(q_{1}\right)},D_{ij}^{\left(q_{2}\right)}\right)$ such that the lower *q*_1_^th^ and the upper *q*_2_^th^ were not symmetrical.

Second, whether the actual value of the extreme quantiles in differences in indicator responses is interpreted as evidence for a distinction between indicators and the facets they are assessing will depend on various aspects of the nature of the indicators themselves. The interpretation of the QED matrix will arguably be most straightforward when each of the indicators uses the same response scale with the same anchors. We will consider variations on the QED matrix below that may be applicable even when these conditions are not met. However, even when these conditions are met, whether a given difference in indicator responses is considered sufficiently extreme to constitute evidence for an important distinction across indicator facets will depend on the nature of the scale and of the anchors. In most cases, a 1-point difference would likely not be viewed as constituting much evidence for an especially pertinent distinction. If the indicators themselves were viewed (as is often the case) as reflecting an underlying univariate latent variable subject to error, then a 1-point, or even a 2-point difference, might be routinely expected. When considering whether a particular extreme difference might constitute evidence for a distinction in the facets the indicators reflect, it is important to consider what this difference corresponds to with regard to participant responses. The number of response categories is of course relevant here. A 3-point response difference on a 5-point likert scale would in most cases arguably be stronger evidence for a distinction across indicator facets than would a 3-point response difference on an 11-point likert scale. The interpretation of response differences will also in general be easier when the anchors used for a likert scale are the same across items. This may not always be essential if there are other reasonable grounds for comparison, such as in some well-being assessments in which, while the anchors are different, an 11-point likert scale is uniformly employed [22], 23], and the intuitive 0–10 rating gives some grounds for comparability.

Third, in certain cases, it may be that two indicators are assessing essentially the same facet of the construct but the wording of the item corresponding to one indicator may be considerably stronger, or harder to satisfy or agree to, than that of the other. One might thus observe extreme differences on account of the relative “difficulty” of one indicator as compared with another. Examples in which only one of the quantiles of extreme difference is large in absolute magnitude, and the other small, may thus not constitute much evidence for distinctions in indicator facets. It may rather instead simply arise because one of the items corresponds to a greater level of difficulty than the other. If, however, both of the extreme quantiles are large in magnitude and of opposite signs this cannot then simply be a matter of the relative difficulty of items and would constitute greater evidence of distinctions across the indicators. It should also be noted that it is in principle possible for some indicator *i* say to have a higher mean than another indicator *j*, but for the quantiles of differences to make clear that there is at least *q*% of the sample with considerably higher values for indicator *j* than indicator *i*, even if it is not the case that there are also *q*% of the sample with similarly higher values for indicator *i* than indicator *j*. In such cases, the quantiles of extreme differences would arguably again provide some evidence for distinctions across indicator facets, and could not be attributed simply to the greater difficulty of one of the indicators. More formally, if the mean of *X*_*i*_ is higher than the mean of *X*_*j*_ but $D_{ij}^{\left(q\right)}$ is a large negative value then this may constitute evidence for a distinction across indicator facets; and if the mean of *X*_*j*_ is higher than the mean of *X*_*i*_ but $D_{ij}^{\left(1-q\right)}$ is a large positive value then again this may similarly constitute evidence for a distinction across indicator facets.

Fourth, it is possible that extreme differences for responses between two indicators may arise for an individual not because the indicators correspond to distinct facets of the construct but simply because of greater error and consequent variability in the reporting of one indicator vs. the other. This concern, along with the concern above regarding differences in the difficulty of items, could both be at least partially addressed by first standardizing each of the indicators before computing the QED matrix. This approach is also applicable if the items corresponding to the indicators potentially use different scaling or different anchors. Suppose one were to observe relatively substantial values for both of the extreme quantiles of differences in the standardized indicators. In that case, this would again arguably constitute evidence for distinctions in indicator facets. The strongest possible evidence for such distinctions arguably arises when it is the case that there are relatively substantial values for both of the extreme quantiles of differences in the *unstandardized* indicators, and also for both of the extreme quantiles of differences in the *standardized* indicators.

Fifth, given that the choice of the quantile *q* is relatively arbitrary, yet another variation on the reporting proposed here would be to *a priori* set a difference between indicators that was thought to be sufficiently large so as to provide evidence for a distinction across indicator facets and then to report the proportion of individuals in the sample for which the difference in indicators were at least that large. More formally, for indicators *X*_*i*_ and *X*_*j*_ and specified difference *d* let $p_{ij}^{d}$ denote the proportion of individuals for whom *X*_*i*_ − *X*_*j*_≥*d*. The reporting of $p_{ij}^{d}$ and $p_{ji}^{d}$ would thus also constitute the proportion of individuals for whom the differences in responses of magnitude *d* would have to be due to misinterpretation or misreporting or other forms of error for the observed differences not to constitute evidence for a distinction across indicator facets. The matrix $M_{\mathrm{QEDP}}^{d}$ whose *i-j* entry is given by the bivariate quantities $\left(p_{ij}^{d}\text{,}p_{ji}^{d}\right)$ we will refer to as the QED proportions matrix, and the sum of these two proportions, $p_{ij}^{d}+p_{ji}^{d}$ , might also be presented as a summary. This might likewise be reported both for the unstandardized indicator differences of magnitude *d*, and also for the standardized indicator differences of a particular magnitude expressed in terms of standardized points. For example, one might specify differences of at least *d*=1.65 standardized points. A difference of 1.65 standardized points would correspond to, for example, a value for one indicator at only the 50th percentile, but a value for the other indicator at the 95th percentile; or alternatively, a value for one indicator at only the 20th percentile, but a value for the other indicator at the 80th percentile. These would in most contexts constitute notable differences.

Sixth, the extreme quantiles of differences can be empirically compared to what would be expected in a given factor model. The quantiles of extreme differences matrix could be seen as analogous to the observed covariance matrix, where differences within response patterns are evaluated instead of similarity in response patterns. Suppose a single factor model holds such that *X*_*i*_=*τ*_*i*_ + *λ*_*i*_ *η* + *e*_*i*_ with normally distributed errors *e*_*i*_, then the model implied expected response is $E\left[X_{i}\right]=\tau_{i}$ and we can denote the model implied covariance matrix as $\Sigma \left(\theta \right)$ . The distribution of expected difference then has a known distribution, $E\left[X_{i}-X_{j}\right]=\tau_{i}-\tau_{j}$ , with a known variance, $V\left[X_{i}-X_{j}\right]=V\left[X_{1}\right]+V\left[X_{2}\right]-2Cov\left[X_{1},X_{2}\right]=\left(\lambda_{i}^{2}+\lambda_{j}^{2}-2\lambda_{i}\lambda_{j}\right)V\left[\eta \right]+V\left[e_{i}\right]+V\left[e_{j}\right]$ , which is easily obtained from the solution of the factor model. For standardized indicators under a factor model this variance in the difference is given simply by 2 − 2 × Cor(*X*_1_, *X*_2_). One could then compare the quantiles of the implied distribution of the differences to the observed quantiles of extreme differences. Let $D_{ij}^{\left(q\right)}\left(\theta \right)$ represent the model implied quantile of the difference distribution, then an empirical evaluation can be made between the observed $D_{ij}^{\left(q\right)}$ and the model predicted $D_{ij}^{\left(q\right)}\left(\theta \right)$ . The empirical comparison of $D_{ij}^{\left(q\right)}$ and $D_{ij}^{\left(q\right)}\left(\theta \right)$ mirrors what is currently common practice in factor analysis of comparing the observed covariance matrix *S* with the model implied covariance matrix Σ(*θ*) in model fit evaluation [24]. The proposed evaluation of extreme quantiles is similar, but with the focus on extreme differences, instead of similarities, in responses.

One might also similarly consider this comparison for both of the *q*th and (1-*q*)^th^ quantiles and thus, for example compare $D_{ij}^{\left(1-q\right)}-D_{ij}^{\left(q\right)}$ and $D_{ij}^{\left(1-q\right)}\left(\theta \right)-D_{ij}^{\left(q\right)}\left(\theta \right)$ . When the observed quantiles are more extreme than the factor model implies, then this is arguably additional evidence of conceptual distinctions. This can potentially provide helpful benchmarking concerning how much more (or less) extreme are the observed differences in indicators at quantiles *q* and (1-*q*) than what one might expect from a factor model. It should, however, be noted that even if these differences in quantiles correspond to what might be expected from a univariate factor model, the distinctions amongst the various indicators might nevertheless still be quite relevant and correspond to very different potential causal effects of the underlying phenomena to which the indicators correspond [6], 7]. Another helpful benchmark might be constituted by what these expected differences in quantiles might be for two independent random variables. If such variables are standardized then the difference would have variance 2 and the 2.5th and 97.5th quantiles of the difference would be −2.77 and 2.77 respectively, with the difference between these quantiles being 5.54.

Likewise, for the standardized QED proportions matrix, for two indicators that had a correlation of 0.75 under a univariate factor model, the expected total proportion of the population with differences in standardized scores exceeding 1.65, i.e. $\left(p_{ij}^{1.65}+p_{ji}^{1.65}\right)$ , is less than 2 %. For two indicators that had a correlation of 0.5, this expected proportion is slightly less 10 %. For two independent variables, this expected proportion is about 24 %. These might likewise constitute helpful benchmarking values.

Seventh and finally, in the discussion above, we have focused the analytic considerations on potential evidence for distinctions across construct facets that the individual indicators may reflect. In some cases, certain scales and assessments are hierarchically ordered into domains or even subdomains sometimes supposedly corresponding to facets or subfacets. In such cases the approach we have described concerning evidence for distinctions across indicator facets could be employed instead to the facets of the construct phenomenon that correspond to specific domains or subdomains, rather than to the indicators. One could replace the individual indicators with the average value of the indicators within the domains or within the subdomains and then report the QED matrix, or the variations above, for these domain or subdomain scores to potentially provide evidence for distinctions across domain or subdomain facets. Because the domain and subdomain scores are themselves averages, the thresholds for what constitutes an extreme difference would arguably be smaller for domains and subdomains than for indicators, and likewise smaller for domains than for the subdomains. When appropriate, the QED matrix could again in principle be reported both for unstandardized and for standardized domain and subdomain scores. Once again, however, what constitutes an extreme difference in domain and subdomain scores so as to provide evidence for a distinction in domain and subdomain facets will depend on the context and the nature of the response scales being employed.

We will now illustrate these various approaches in a couple of empirical examples.

## Example 1. satisfaction with life scale

In what follows we will report and comment on the QED matrix and related matrices for unstandardized and standardized indicators of Diener et al.’s [25] Satisfaction with Life Scale. We use data from the 2020 wave of Health and Retirement Study. The Satisfaction with Life Scale is a well-established measure of self-evaluated life quality that has been used extensively in comparative international studies; the paper introducing the measure has been cited over 40,000 times. The scale consists of five items: “In most ways my life is close to ideal”; “The conditions of my life are excellent”; “I am satisfied with my life”; “So far, I have gotten the important things I want in life”; and “If I could live my life again, I would change almost nothing”. A 7-point likert scale is employed for each item: 1=strongly disagree; 2=disagree; 3=slightly disagree; 4=neither agree nor disagree; 5=slightly agree; 6=agree; 7=strongly agree. The specific items were chosen and the scale was evaluated using factor analytic methods. The scale has been documented to have very good psychometric properties: Cronbach’s alpha is high and a single underlying factor seems to explain a considerable proportion of the shared variance across item responses [25], 26]. We employ data from a sample of 4,257 respondents in the 2020 wave of the Health and Retirement Study. These data are openly available online at (https://hrsdata.isr.umich.edu/data-products/public-survey-data). The correlation matrix for the five indicators, along with their means and standard deviations, are reported in Table 1.

**Table 1:** Correlation matrix for the satisfaction with life scale.

Item	(1)	(2)	(3)	(4)	(5)
	**Observed correlations**				
Life is close to ideal (1)		0.75	0.68	0.56	0.46
Life conditions are excellent (2)	0.75		0.75	0.60	0.47
Satisfied with life (3)	0.68	0.75		0.70	0.49
Have important things in life (4)	0.56	0.60	0.70		0.53
Change none if lived life over (5)	0.46	0.47	0.49	0.53	
Mean	4.90	4.89	5.44	5.55	4.47
SD	1.78	1.81	1.71	1.61	2.02

The QED matrix for *q*=2.5 for the unstandardized indicators is reported in Table 2.

**Table 2:** Quantiles of extreme difference matrix (q=2.5) for the unstandardized satisfaction with life scale indicators.

Item	(1)	(2)	(3)	(4)	(5)
Life is close to ideal (1)		(−3, 3)	(−4, 2)	(−5, 2)	(−4, 5)
Life conditions are excellent (2)	(−3, 3)		(−4, 2)	(−4, 2)	(−4, 5)
Satisfied with life (3)	(−2, 4)	(−2, 4)		(−3, 3)	(−3, 5)
Have important things in life (4)	(−2, 5)	(−2, 4)	(−3, 3)		(−2, 5)
Change none if lived life over (5)	(−5, 4)	(−5, 4)	(−5, 3)	(−5, 2)	

The average difference between quantiles is 7.0.

Note that a 3-point difference would correspond to differences in responses of “Neither agree nor disagree” vs. “strongly agree” (or “strongly disagree”), or alternatively to differences in responses between “slightly disagree” vs. “agree.” There are at least 2.5 % of the sample (i.e. at least 4,257 × 0.025=106 individuals) who report such differences in their responses, with their response to item (1) “In most ways my life is close to ideal” considerably higher than to item (2) “The conditions of my life are excellent.” And conversely also at least 2.5 % (i.e. at least 106 individuals) whose response to item (2) is considerably higher than to item (1). There may thus, for example, be a number who recognize the conditions of their life as excellent, but do not believe it is “close to ideal” (e.g. perhaps because “the ideal” is yet so much greater). Conversely, however, it seems there are a number of people who do say that their life is “close to ideal,” but would not affirm that the conditions of their life are excellent, perhaps because, for example, the challenging conditions have led to important growth or because although the overall conditions of life are not excellent, the most important things are, etc. Identification of such individuals who report these differences may motivate further interviews and qualitative work on understanding how and why these participants responded so differently to the various indicators.

The quantiles of extreme differences are even more dramatic if we compare indicators (1) and (5) for example. There are again at least 106 of the 4,257 respondents who on the 1–7 likert scale respond to item (1) “In most ways my life is close to ideal” 5 points higher than to item (5) “If I could live my life again, I would change almost nothing,” but also at least 106 respondents who give a response to item (5) that is 4 points higher than to item (1). These indicators are clearly picking up different facets of satisfaction with life. Note that we can arguably make such claims that there are conceptually distinct facets of satisfaction with life in spite of the fact that a single factor seems to explain most of the shared variation across indicators [25], 26].

Suppose we want to examine these quantiles of extreme differences through a slightly different lens, acknowledging the differing means and standard deviations of the various indicators. In that case, we can also report the QED matrix for *q*=2.5 for the standardized indicators, which is given in Table 3.

**Table 3:** Quantiles of extreme difference (q=2.5) for the standardized satisfaction with life scale indicators.

Item	(1)	(2)	(3)	(4)	(5)
Life is close to ideal (1)		(−1.68, 1.66)	(−1.95, 1.51)	(−2.47, 1.69)	(−2.38, 2.34)
Life conditions are excellent (2)	(−1.66, 1.68)		(−1.92, 1.42)	(−1.94, 1.70)	(−2.35, 2.33)
Satisfied with life (3)	(−1.51, 1.95)	(−1.42, 1.92)		(−1.74, 1.87)	(−2.18, 2.13)
Have important things in life (4)	(−1.69, 2.47)	(−1.70, 1.94)	(−1.87, 1.74)		(−1.71, 2.12)
Change none if lived life over (5)	(−2.34, 2.38)	(−2.33, 2.35)	(−2.13, 2.18)	(−2.12, 1.71)	

The average difference between quantiles is 3.91.

From this standardized QED matrix we could see that for at least 2.5 % of the sample (i.e. for at least 106 of the 4,257 respondents), the participants response to item (1) “In most ways my life is close to ideal” is 1.68 standardized points higher than to item (2) “The conditions of my life are excellent.” A difference of 1.68 standardized points would correspond to, for example, a value for indicator 2 at only the 50th percentile, but a value for indicator 1 at the 95th percentile; or alternatively, a value for indicator 2 at only the 20th percentile, but a value for indicator 1 at the 80th percentile. These are of course very notable differences.

We can also again see from the standardized QED matrix the relatively extreme values in the 2.5th and 97.5th quantiles of the differences in responses for indicators (1) and (5), corresponding to −2.38 and 2.34 standardized points respectively (with a difference in these quantiles of $D_{ij}^{\left(1-q\right)}-D_{ij}^{\left(q\right)}=2.34-\left(-2.38\right)=4.72$ . As noted above, we could compare these observed quantiles to the expected quantiles of the difference under a univariate factor model, which, with standardized indicators, would have a variance of $V\left[X_{i}\right]+V\left[X_{j}\right]-2cor\left(X_{i},X_{j}\right)=2-2cor\left(X_{i}X_{j}\right)=2-2\left(0.46\right)=1.08$ . The 2.5th and 97.5th quantiles of a normal variable with variance 1.08 are −2.04 and 2.04, respectively (with a difference in these expected quantiles of $D_{ij}^{\left(q\right)}\left(\theta \right)-D_{ij}^{\left(q\right)}\left(\theta \right)=2.04-\left(-2.04\right)=4.08$ . We see then that the observed quantiles of relatively extreme differences are larger than what one might anticipate from a univariate factor model. As noted above, the 2.5th and 97.5th quantiles of the difference of two completely independent standard normal variables are −2.77 and 2.77 (with a difference in these quantiles of 5.54). Similar analyses for all other pairs of indicators are given in the online Supplement (Supplementary Tables S1–S3).

As noted above, we might also instead consider reporting for each pair of indicators the proportions of the difference that exceed a certain threshold. In the notation given above this QED proportions matrix was given by entries $\left(p_{ij}^{d}\text{,}p_{ji}^{d}\right)$ where $p_{ij}^{d}$ is the proportion for whom *X*_*i*_ − *X*_*j*_≥*d*, and report also in bold the sum $\left(p_{ij}^{d}+p_{ji}^{d}\right)$ . This QED proportions matrix for d=3 for the Satisfaction with Life Scale data is given in Table 4.

**Table 4:** QED proportions for the unstandardized satisfaction with life scale indicators (d=3).

Item	(1)	(2)	(3)	(4)	(5)
Life is close to ideal (1)		**7.1 %** (3.5 %, 3.5 %)	**10.0 %** (1.7 %, 8.3 %)	**13.8 %** (2.1 %, 11.7 %)	**21.7 %** (15.0 %, 6.7 %)
Life conditions are excellent (2)	**7.1 %** (3.5 %, 3.5 %)		**8.4 %** (1.1 %, 7.3 %)	**13.6 %** (1.9 %, 11.7 %)	**21.1 %** (14.7 %, 6.4 %)
Satisfied with life (3)	**10.0 %** (8.3 %, 1.7 %)	**8.4 %** (7.3 %, 1.1 %)		**7.4 %** (2.6 %, 4.8 %)	**23.8 %** (20.6 %, 3.2 %)
Have important things in life (4)	**13.8 %** (11.7 %, 2.1 %)	**13.6 %** (11.7 %, 1.9 %)	**7.4 %** (4.8 %, 2.6 %)		**22.4 %** (20.6 %, 1.8 %)
Change none if lived life over (5)	**21.7 %** (6.7 %, 15.0 %)	**21.1 %** (6.4 %, 14.7 %)	**23.8 %** (3.2 %, 20.6 %)	**22.4 %** (1.8 %, 20.6 %)	

The bolded number is the sum of the two proportions.

From this, we see that responses to items 1, 2, 3, and 4 are at least 3 points higher than item responses to item 5 for at approximately 15.0 , 14.7, 20.6, and 20.6 % of the sample. In contrast, response to item 5 are reported least 3 points higher than items 1, 2, 3, and 4 for only 6.7 %, 6.4 %, 3.2 %, and 1.8 % of the sample respectively.

Finally, Table 5 reports the QED proportions matrix for the standardized indicators with the proportions whose differences in standardized indicators are greater than *d*=1.65 points. As examples, we see that standardized scores on item 4 were at least 1.65 standardized points higher than standardized responses to item 5 for approximately 5.0 % of the sample. In contrast, standardized responses to item 5 were 1.65 standardized points higher than on item 4 for approximately 2.9 % of the sample. The sum of proportions exceeding +/−1.65 differences in standardized points for items 1 and 2 is approximately 6.6 % of the sample whereas the combined proportion of extreme differences was much greater for the standardized responses to items 1 and 5 at approximately 13.5 % of the sample.

**Table 5:** QED proportions for the standardized satisfaction with life scale indicators (d=1.65).

Item	(1)	(2)	(3)	(4)	(5)
Life is close to ideal (1)		**6.6 %** (3.1 %, 3.5 %)	**5.0 %** (1.7 %, 3.2 %)	**9.4 %** (3.0 %, 6.4 %)	**13.5 %** (6.9 %, 6.7 %)
Life conditions are excellent (2)	**6.6 %** (3.5 %, 3.1 %)		**4.3 %** (1.1 %, 3.2 %)	**8.7 %** (2.8 %, 5.9 %)	**13.3 %** (6.9 %, 6.4 %)
Satisfied with life (3)	**5.0 %** (3.2 %, 1.7 %)	**4.3 %** (3.2 %, 1.1 %)		**7.1 %** (2.6 %, 4.5 %)	**8.9 %** (4.7 %, 4.2 %)
Have important things in life (4)	**9.4 %** (6.4 %, 3.0 %)	**8.7 %** (5.9 %, 2.8 %)	**7.1 %** (4.5 %, 2.6 %)		**7.9 %** (5.0 %, 2.9 %)
Change none if lived life over (5)	**13.5 %** (6.7 %, 6.9 %)	**13.3 %** (6.4 %, 6.9 %)	**8.9 %** (4.2 %, 4.7 %)	**7.9 %** (2.9 %, 5.0 %)	

The bolded number is the sum of the two proportions.

We provide code to implement these various approaches and output these matrices in the online Supplement and through the newly introduced **R** package *qedmetrics* (https://doi.org/10.17605/osf.io/pqwb2).

## Example 2. The comprehensive measure of meaning

As a second example, we will consider data from Padgett et al. [27] employing responses from 4,058 students at the University of British Columbia, Canada concerning the Comprehensive Measure of Meaning assessment [27], 28]. The Comprehensive Measure of Meaning assessment measure is constituted by 21 items which are categorized into three domains: coherence, significance, and direction. The 3 domains are further divided into 7 distinct subdomains, each of which has three items: coherence is divided global coherence (CG) vs. individual coherence (CI); significance is divided into subjective significance (SS) vs. objective significance (SO); and direction is divided into mission (DM), purposes (DP) and goals (DG). In the study, a 7-point likert response scale was employed for each item, once again with anchors ranging from 1=strongly disagree to 7=strongly agree. We will use this example to illustrate the QED matrix with indicators nested within subdomains which are then further nested within domains.

The QED matrix for *q*=2.5 for the unstandardized indicators is reported in Table 6.

**Table 6:** Quantiles of extreme difference matrix for the unstandardized indicators of comprehensive measure of meaning.

	CG1	CG2	CG3	CI1	CI2	CI3	SS1	SS2	SS3	SO1	SO2	SO3	DM1	DM2	DM3	DP1	DP2	DP3	DG1	DG2	DG3
CG1		(−2, 2)	(−4, 2)	(−3, 2)	(−4, 2)	(−4, 2)	(−4, 3)	(−5, 3)	(−4, 3)	(−4, 3)	(−4, 4)	(−4, 4)	(−3, 3)	(−4, 3)	(−4, 3)	(−4, 2)	(−4, 3)	(−5, 2)	(−4, 3)	(−5, 2)	(−5, 2)
CG2	(−2, 2)		(−4, 2)	(−3, 2)	(−4, 2)	(−4, 2)	(−4, 3)	(−5, 2)	(−3, 3)	(−4, 3)	(−4, 3)	(−4, 4)	(−3, 3)	(−4, 3)	(−4, 3)	(−4, 2)	(−4, 3)	(−5, 2)	(−5, 3)	(−5, 2)	(−5, 2)
CG3	(−2, 4)	(−2, 4)		(−2, 3)	(−3, 2)	(−3, 3)	(−3, 4)	(−4, 3)	(−3, 4)	(−4, 3)	(−3, 4)	(−3, 4)	(−2, 4)	(−3, 4)	(−3, 4)	(−3, 3)	(−3, 4)	(−4, 3)	(−4, 3)	(−4, 2)	(−4, 3)
CI1	(−2, 3)	(−2, 3)	(−3, 2)		(−4, 2)	(−4, 2)	(−3, 3)	(−4, 2)	(−3, 3)	(−4, 3)	(−3, 4)	(−3, 4)	(−3, 3)	(−3, 3)	(−4, 3)	(−4, 2)	(−3, 3)	(−4, 2)	(−4, 3)	(−5, 2)	(−5, 2)
CI2	(−2, 4)	(−2, 4)	(−2, 3)	(−2, 4)		(−2, 3)	(−2, 4)	(−3, 3)	(−2, 4)	(−3, 3)	(−2, 4)	(−2, 4)	(−2, 4)	(−3, 4)	(−3, 4)	(−3, 4)	(−2, 4)	(−3, 3)	(−3, 4)	(−4, 3)	(−4, 3)
CI3	(−2, 4)	(−2, 4)	(−3, 3)	(−2, 4)	(−3, 2)		(−3, 4)	(−4, 3)	(−2, 4)	(−3, 3)	(−3, 4)	(−2, 4)	(−2, 4)	(−3, 4)	(−3, 4)	(−3, 3)	(−3, 4)	(−4, 3)	(−4, 3)	(−4, 2)	(−4, 3)
SS1	(−3, 4)	(−3, 4)	(−4, 3)	(−3, 3)	(−4, 2)	(−4, 3)		(−4, 2)	(−2, 3)	(−4, 3)	(−3, 3)	(−3, 3)	(−3, 4)	(−3, 4)	(−3, 3)	(−3, 2)	(−3, 3)	(−4, 2)	(−4, 3)	(−4, 2)	(−4, 2)
SS2	(−3, 5)	(−2, 5)	(−3, 4)	(−2, 4)	(−3, 3)	(−3, 4)	(−2, 4)		(−2, 4)	(−3, 3)	(−2, 4)	(−2, 4)	(−2, 4)	(−3, 4)	(−3, 4)	(−3, 3)	(−3, 4)	(−4, 3)	(−4, 4)	(−4, 3)	(−4, 3)
SS3	(−3, 4)	(−3, 3)	(−4, 3)	(−3, 3)	(−4, 2)	(−4, 2)	(−3, 2)	(−4, 2)		(−4, 2)	(−3, 3)	(−3, 3)	(−3, 3)	(−3, 3)	(−4, 3)	(−4, 2)	(−3, 3)	(−4, 2)	(−4, 3)	(−5, 2)	(−4, 2)
SO1	(−3, 4)	(−3, 4)	(−3, 4)	(−3, 4)	(−3, 3)	(−3, 3)	(−3, 4)	(−3, 3)	(−2, 4)		(−2, 4)	(−2, 4)	(−2, 4)	(−3, 4)	(−3, 4)	(−3, 3)	(−3, 4)	(−4, 3)	(−3, 3)	(−4, 2)	(−4, 3)
SO2	(−4, 4)	(−3, 4)	(−4, 3)	(−4, 3)	(−4, 2)	(−4, 3)	(−3, 3)	(−4, 2)	(−3, 3)	(−4, 2)		(−3, 3)	(−3, 3)	(−4, 4)	(−4, 3)	(−4, 3)	(−4, 3)	(−4, 2)	(−4, 3)	(−5, 2)	(−5, 2)
SO3	(−4, 4)	(−4, 4)	(−4, 3)	(−4, 3)	(−4, 2)	(−4, 2)	(−3, 3)	(−4, 2)	(−3, 3)	(−4, 2)	(−3, 3)		(−3, 3)	(−4, 3)	(−4, 3)	(−4, 2)	(−4, 3)	(−4, 2)	(−4, 2)	(−5, 1)	(−5, 2)
DM1	(−3, 3)	(−3, 3)	(−4, 2)	(−3, 3)	(−4, 2)	(−4, 2)	(−4, 3)	(−4, 2)	(−3, 3)	(−4, 2)	(−3, 3)	(−3, 3)		(−3, 2)	(−4, 2)	(−4, 2)	(−3, 2)	(−4, 2)	(−4, 2)	(−5, 2)	(−5, 2)
DM2	(−3, 4)	(−3, 4)	(−4, 3)	(−3, 3)	(−4, 3)	(−4, 3)	(−4, 3)	(−4, 3)	(−3, 3)	(−4, 3)	(−4, 4)	(−3, 4)	(−2, 3)		(−3, 2)	(−4, 2)	(−3, 3)	(−4, 2)	(−4, 3)	(−5, 2)	(−5, 2)
DM3	(−3, 4)	(−3, 4)	(−4, 3)	(−3, 4)	(−4, 3)	(−4, 3)	(−3, 3)	(−4, 3)	(−3, 4)	(−4, 3)	(−3, 4)	(−3, 4)	(−2, 4)	(−2, 3)		(−3, 1)	(−3, 3)	(−4, 2)	(−4, 3)	(−5, 2)	(−4, 2)
DP1	(−2, 4)	(−2, 4)	(−3, 3)	(−2, 4)	(−4, 3)	(−3, 3)	(−2, 3)	(−3, 3)	(−2, 4)	(−3, 3)	(−3, 4)	(−2, 4)	(−2, 4)	(−2, 4)	(−1, 3)		(−2, 3)	(−3, 2)	(−3, 3)	(−4, 2)	(−4, 3)
DP2	(−3, 4)	(−3, 4)	(−4, 3)	(−3, 3)	(−4, 2)	(−4, 3)	(−3, 3)	(−4, 3)	(−3, 3)	(−4, 3)	(−3, 4)	(−3, 4)	(−2, 3)	(−3, 3)	(−3, 3)	(−3, 2)		(−4, 2)	(−4, 2)	(−5, 1)	(−4, 2)
DP3	(−2, 5)	(−2, 5)	(−3, 4)	(−2, 4)	(−3, 3)	(−3, 4)	(−2, 4)	(−3, 4)	(−2, 4)	(−3, 4)	(−2, 4)	(−2, 4)	(−2, 4)	(−2, 4)	(−2, 4)	(−2, 3)	(−2, 4)		(−2, 3)	(−3, 2)	(−3, 3)
DG1	(−3, 4)	(−3, 5)	(−3, 4)	(−3, 4)	(−4, 3)	(−3, 4)	(−3, 4)	(−4, 4)	(−3, 4)	(−3, 3)	(−3, 4)	(−2, 4)	(−2, 4)	(−3, 4)	(−3, 4)	(−3, 3)	(−2, 4)	(−3, 2)		(−4, 1)	(−3, 2)
DG2	(−2, 5)	(−2, 5)	(−2, 4)	(−2, 5)	(−3, 4)	(−2, 4)	(−2, 4)	(−3, 4)	(−2, 5)	(−2, 4)	(−2, 5)	(−1, 5)	(−2, 5)	(−2, 5)	(−2, 5)	(−2, 4)	(−1, 5)	(−2, 3)	(−1, 4)		(−2, 3)
DG3	(−2, 5)	(−2, 5)	(−3, 4)	(−2, 5)	(−3, 4)	(−3, 4)	(−2, 4)	(−3, 4)	(−2, 4)	(−3, 4)	(−2, 5)	(−2, 5)	(−2, 5)	(−2, 5)	(−2, 4)	(−3, 4)	(−2, 4)	(−3, 3)	(−2, 3)	(−3, 2)	

CG, coherence global; CI, coherence individual; SS, significance subjective; SO, significance objective; DM, direction mission; DP, direction purpose; DG, direction goals.

Once again, a 3-point difference would correspond to differences in responses of “Neither agree nor disagree” vs. “strongly agree” (or “strongly disagree”), or alternatively to differences in responses between “slightly disagree” vs. “agree.” Many of these pairs of indicators manifest a 3-point difference in at least one of the 2.5th or 97.5th quantiles, or, in many cases, in both. This is also often the case for pairs of indicators even within subdomains (with at least one of these two quantiles constituting a difference of at least 3 points), though there are exceptions. The first and second indicators in the global coherence subdomain only manifest differences of 2 points at the 2.5th or 97.5th quantiles There is thus perhaps less evidence that these two indicators represent distinct facets of global coherence. The two corresponding items are: “I have a clear understanding of the ultimate meaning of life” (CG1) and “The meaning of life in the world around us is evident to me” (CG2). With regard to the first and second indicator in the goals domain (DG), the 2.5th quantile of the difference between the first and second indicator is only −1, though the difference for the 97.5th quantile is 4. In this case, the second goals indicator also has a considerably higher mean, 5.36, than does the first, 4.58. This may be an example of the substantial differences manifest at the 97.5th being due to greater difficulty of the first item rather than the indicators manifesting distinct facets of goals. The two corresponding items are: “In my life I have very clear goals and aims” (DG1) and “I have goals in life that are very important to me” (DG2).

The QED matrix for *q*=2.5 for the seven CMM subdomains is reported in Table 7 and for the three CMM domains in Table 8.

**Table 7:** Quantiles of extreme difference matrix for the sub-domains of the comprehensive measure of meaning.

	CG	CI	SS	SO	DM	DP	DG
CG		(−2.67, 1.33)	(−3.00, 2.33)	(−3.00, 2.67)	(−2.67, 3.00)	(−3.33, 2.00)	(−4.00, 1.67)
CI	(−1.33, 2.67)		(−1.67, 2.33)	(−2.00, 3.00)	(−2.00, 3.00)	(−2.00, 2.00)	(−3.00, 2.00)
SS	(−2.33, 3.00)	(−2.33, 1.67)		(−2.00, 2.33)	(−2.00, 3.00)	(−2.33, 1.67)	(−3.33, 1.74)
SO	(−2.67, 3.00)	(−3.00, 2.00)	(−2.33, 2.00)		(−2.33, 3.00)	(−2.67, 2.00)	(−3.67, 1.67)
DM	(−3.00, 2.67)	(−3.00, 2.00)	(−3.00, 2.00)	(−3.00, 2.33)		(−3.00, 1.33)	(−4.00, 1.33)
DP	(−2.00, 3.33)	(−2.00, 2.00)	(−1.67, 2.33)	(−2.00, 2.67)	(−1.33, 3.00)		(−2.67, 1.33)
DG	(−1.67, 4.00)	(−2.00, 3.00)	(−1.74, 3.33)	(−1.67, 3.67)	(−1.33, 4.00)	(-1.33, 2.67)	

Emphasis (box) around subdomains that are under the same domain. CG, coherence global; CI, coherence individual; SS, significance subjective; SO, significance objective; DM, direction mission; DP, direction purpose; DG, direction goals.

**Table 8:** Quantiles of extreme difference matrix for the domains of the comprehensive measure of meaning.

	Coherence	Significance	Direction
Coherence		(−2.00, +2.17)	(−2.44, +1.67)
Significance	(−2.17, +2.00)		(−2.11, +1.46)
Direction	(−1.67, +2.44)	(−1.46, +2.11)	

The extreme quantiles (2.5 and 97.5 %) for the individual-level differences among domain scores in Table 8 indicate substantial differences for a notable proportion of the sample. There was 2.5 % of the sample (i.e., 101 of the 4,058 individuals) who scored 2.17 points or more higher on coherence than on significance and also 2.5 % of the sample (i.e., 101 of the 4,058 individuals) who conversely scored 2.00 points or more higher on significance than on coherence. Likewise similar substantial differences are apparent in the extreme quantiles comparing other domains, thereby supporting the conceptual distinctions across these domains. Similar evidence for distinctions pertain to the subdomains in Table 7, and, as noted above, in many cases to the individual indicators in Table 6. The average difference between the two extreme quantiles of differences in Table 8 for the domains, 3.98, is smaller than that for the subdomains, 4.92, in Table 7, which is in turn smaller than that for the individual indicators, 6.42, in Table 6.

Standardized QED and standardized QED proportions matrices for indicators, subdomains, and domains are presented in the online Supplement (Supplementary Tables S4–S9).

## Discussion

In this paper, we have considered using outer quantiles corresponding to relatively extreme differences between indicators to provide evidence for distinctions in the facets of the construct phenomenon to which these indicators (or indicator means) correspond. We have discussed various criteria, and points of interpretation and further caveats, about using the matrix reporting these quantiles of extreme differences for such purposes. Of course, simply because a valid distinction can be drawn does not imply that the distinction is necessarily relevant in a given context. It may be the case that although certain facets of a construct phenomenon are distinct in the sense that one may be present without the other, these different facets might nevertheless also still functionally relate to outcomes of interest in a similar way. It would then remain to be examined whether the various facets that were distinguished related differently to outcomes of interest in their effects, or whether these different facets had potentially different causes. The answers to such questions cannot be discerned simply by examining relations of the indicators with one another. Evidence for different causes of distinct indicators, and the facets of the construct phenomenon to which they correspond, could potentially be evaluated in randomized trials of interventions designed to alter the construct phenomenon of interest, but then examining the indicators one by one [29]. Distinct effects of the various indicator facets on different outcomes can likewise be examined using longitudinal data [6], 7], 9]. Important distinctions between indicators can thus in principle be established either on the grounds of differential causes or effects, or, as in this paper, by evidence that there are individuals for whom one particular facet of the construct phenomenon is present and another is relatively absent. This latter approach, considered here, might lead to and potentially motivate the former.

As noted above, the relevant thresholds for the QED matrix depend on context – on the number of response categories on a likert scale, and, for example, on the associated anchors or labels. This is perhaps especially relevant if the QED proportions matrix is being reported as these proportions will of course vary potentially quite dramatically depending on the threshold that is set. One might propose rules of thumb such as reporting on proportions of indicators differences of at least 2 for a 5-point likert scale, or of at least 3 for a 7-point likert scale. Such differences would correspond to a change from the mid-point of the scale to its most extreme point. However, whether such differences are what is most relevant may depend on context. On many 11-point likert scales assessed 0–10 in the well-being literature, much of the 0–4 or 0–5 range goes unused, at least in Western contexts. Although there are 11 response categories, it may be more appropriate in this case, for example, to use a 3 point difference as the threshold rather than say a 5-point difference. Reporting the QED proportions matrix for the standardized indicators may simplify matters with some of these nuances but then may also be more difficult to interpret in terms of meaningful response categories. As noted above, maximal insight may arise from examining both standardized and unstandardized QED and QED proportion matrices, and possibly using multiple quantiles or thresholds. This would of course be more than is reasonable to report in many papers but online Supplementary materials will often allow for such more extensive reporting.

The approach of examining quantiles corresponding to extreme difference in indicators might also be of interest, not only in identifying indicators that may correspond to conceptually distinct facets, but also in identifying groups of individuals for whom these indicators, and their distinct facets, are notably different. This may be of interest in its own right. For example, it may be interesting to better understand, and to provide descriptive reporting on, those with higher levels of cognitive coherence but lower level of direction and purpose, or high levels of direction and purpose and low levels of cognitive coherence. Descriptive analyses of the demographic characteristics of such groups may be of interest, as may be examining the response patterns of such individuals to the whole set of the various indicators. Further longitudinal analyses could potentially give rise to insight as to what antecedents might causally bring about individuals manifesting such response patterns. It might further be of interest to consider these groups of individuals in various subgroup analyses in either intervention studies or observational analyses. Such subgroups might also constitute interesting subpopulations on which to undertake further qualitative work to gain greater insight into how certain facets of a phenomenon may be present while others absent. The insights that might emerge from these various approaches could constitute an interesting direction for future research. The use of the QED matrix approach we have described here could further facilitate such research.

As noted repeatedly above, what precisely is reported will depend on the context – both on the context of the nature of the underlying construct phenomenon, and the context of the nature of the assessment items and anchors (and of course also on the space available in a paper). While we believe the QED matrices will often be quite informative when interpreted within the context of the specific anchors on a likert scale, it can be more difficult to understand and interpret these across a range of different QED entries across the entire matrix. The QED proportions may well be easier to make sense of and interpret at a first glance. We would thus propose, when space for presentation and discussion is limited, perhaps presenting the QED proportions matrix for the standardized indicators (using say standardized differences of 1.65 standardized points as a threshold) as a primary analysis, and then perhaps reporting QED matrices and QED proportions for unstandardized indicators in an online Supplement.

The approach we have described has various connections to other work and methods for trying to provide evidence of discrimination of factors. An influential article by Fornell and Larcker [19] described the need to provide evidence of discrimination, or differences between latent variables. As noted above, this is often described as the topic of “discriminant validity.” For example, one popular rule is “the average variance extracted should be larger than the shared variance between factors.” This is done in practice by comparing the average squared standardized factor loading with the squared correlation between factors. If the ratio of these two quantities is greater than one, then this is supposedly evidence of discriminant validity. The rationale is that the factors should explain more variance in the items than the factors explain of each other. The approach we have described here arguably provides a more transparent approach to discriminant validity, and one that it not reliant on an assumption of scientifically meaningful latent factors. Rather, the approach we have presented here is grounded in notions, central to philosophy [21], that distinctions can clearly be established by identifying individual instances in which one phenomenon is present, and the other is not.

We thus believe the reporting of these QED matrices may be of interest, as noted above, both for the purpose of identifying relevant distinctions across indicators, and for the purpose of identifying groups of individuals for whom one facet of a construct phenomenon is present while another is absent. The QED matrix can thus provide a helpful supplement to more traditional correlation matrices, and the analytic approaches based upon them such as factor analysis, which are more oriented towards identifying similarities across indicators, averaging over individuals in the population. We hope the potential adoption of our proposed supplement to psychometric reporting may yield important insights as we seek to better understand the psychosocial phenomena, and relevant distinctions within such phenomena, and their corresponding assessments.

## Supplementary Material

Supplementary Material Details
