# Supplementary material for: The quantiles of extreme differences matrix for evaluating discriminant validity
Source: Epidemiol Methods. 2025 Aug 25;14(1):20250006. doi: 10.1515/em-2025-0006 (PMC12372585; doi:10.1515/em-2025-0006)
Supplement: Supplementary file 1 — Supplementary Material Details [file j_em-2025-0006_suppl_001.docx]

**Online Supplement**

We report here a comparison of the observed quantiles of extreme differences for the Satisfaction with Life Scale (Diener et al., 1985) standardized indicators with the expected quantiles under a univariate latent factor model. The observed quantiles of extreme differences for the standardized indicators was given in the text in Table 3 and is repeated here as Supplementary Table S1.

*Supplementary Table S1. Quantiles of Extreme Difference (q=2.5) for the Standardized Satisfaction with Life Scale Indicators*

| Item | (1) | (2) | (3) | (4) | (5) |
| --- | --- | --- | --- | --- | --- |
| Life is close to ideal (1) |  | (-1.68, 1.66) | (-1.95, 1.51) | (-2.47, 1.69) | (-2.38, 2.34) |
| Life conditions are excellent (2) | (-1.66, 1.68) |  | (-1.92, 1.42) | (-1.94, 1.70) | (-2.35, 2.33) |
| Satisfied with life (3) | (-1.51, 1.95) | (-1.42, 1.92) |  | (-1.74, 1.87) | (-2.18, 2.13) |
| Have important things in life (4) | (-1.69, 2.47) | (-1.70, 1.94) | (-1.87, 1.74) |  | (-1.71, 2.12) |
| Change none if lived life over (5) | (-2.34, 2.38) | (-2.33, 2.35) | (-2.13, 2.18) | (-2.12, 1.71) |  |

Note: The average difference between quantiles is 3.91.

The expected quantiles under a univariate factor model $X_{i}=\lambda_{i}\eta+e_{i}$ for the standardized indicators with normally distributed errors $e_{i}$, can be obtained, as in the text, by noting that these differences would have mean 0 and variance known variance, $V\left[ X_{i}-X_{j} \right]=V\left[ X_{1} \right]+V\left[ X_{2} \right]-2Cov\left[ X_{1},X_{2} \right]=\left( \lambda_{i}^{2}+\lambda_{j}^{2}-2\lambda_{i}\lambda_{j} \right)V\left[ \eta\right]+V\left[ e_{i} \right]+V\left[ e_{j} \right]$. For standardized indicators under the factor model this variance in the difference can also be expressed simply by $2-2\times Cor(X_{1},X_{2})$. Let $D_{ij}^{(q)}\left( \theta\right)$ represent the model implied quantile of the difference distribution. For the Satisfaction with Life data these expected quantiles are given in Supplementary Table S2.

*Supplementary Table S2. Model implied quantiles (q=0.025, q=0.975) under a 1-factor model.*

| Item | (1) | (2) | (3) | (4) | (5) |
| --- | --- | --- | --- | --- | --- |
| Life is close to ideal (1) |  | (-1.51, 1.51) | (-1.49, 1.49) | (-1.74, 1.74) | (-2.01, 2.01) |
| Life conditions are excellent (2) | (-1.51, 1.51) |  | (-1.36, 1.36) | (-1.65, 1.65) | (-1.95, 1.95) |
| Satisfied with life (3) | (-1.49, 1.49) | (-1.36, 1.36) |  | (-1.63, 1.63) | (-1.95, 1.95) |
| Have important things in life (4) | (-1.74, 1.74) | (-1.65, 1.65) | (-1.63, 1.63) |  | (-2.09, 2.09) |
| Change none if lived life over (5) | (-2.01, 2.01) | (-1.95, 1.95) | (-1.95, 1.95) | (-2.09, 2.09) |  |

One might then compare the observed differences in the *q*^th^ and (1-*q*)^th^ quantiles i.e. $D_{ij}^{(1-q)}-D_{ij}^{(q)}$ from Supplementary Table S1, to the expected differences in quantiles under the univariate factor model, $D_{ij}^{(1-q)}\left( \theta\right)-D_{ij}^{(q)}\left( \theta\right)$, and this comparison is given for all pairs of indicators in Supplementary Table S3. Note that other relevant benchmarks here are 0, which would be the difference in quantiles if the indicators were identical and also 5.54, which would correspond to the difference in quantiles for two independently distributed standard normal variables.

Supplementary Table S3. Benchmarking of Standardized QED Matrix Quantile Differences (first entry) with the Expected Difference under a Univariate Factor Model (second entry)

| Item | (1) | (2) | (3) | (4) | (5) | Loading |
| --- | --- | --- | --- | --- | --- | --- |
| Life is close to ideal (1) |  | [3.3, 3.0] | [3.5, 3.0] | [4.2, 3.5] | [4.7, 4.0] | 0.81 |
| Life conditions are excellent (2) | [3.3, 3.0] |  | [3.3, 2.7] | [3.6, 3.3] | [4.7, 3.9] | 0.87 |
| Satisfied with life (3) | [3.5, 3.0] | [3.3, 2.7] |  | [3.6, 3.3] | [4.3, 3.9] | 0.87 |
| Have important things in life (4) | [4.2, 3.5] | [3.6, 3.3] | [3.6, 3.3] |  | [3.8, 4.2] | 0.75 |
| Change none if lived life over (5) | [4.7, 4.0] | [4.7, 3.9] | [4.3, 3.9] | [3.8, 4.2] |  | 0.58 |

Note that in almost instances the observed difference in quantiles exceed what would be expected for a univariate factor model. The one exception concerns indicators 4 and 5 in which the observed difference in quantiles is less than what would be expected from a univariate factor model.

We now turn to our second example on the Comprehensive Measure of Meaning. Standardized QED matrices for domains, subdomains and indicators are presented in the Supplementary Tables S4-S6.

Supplemental Table S4. QED for standardized CMM domain scores

| Domain | (1) | (2) | (3) |
| --- | --- | --- | --- |
| Coherence (1) |  | (-1.68, 1.85) | (-1.80, 1.72) |
| Significance (2) | (-1.85, 1.68) |  | (-1.66, 1.53) |
| Direction (3) | (-1.72, 1.80) | (-1.53, 1.66) |  |

Note: The average difference between quantiles is 3.41.

Supplemental Table S5. QED for standardized CMM subdomain scores

| Item | (1) | (2) | (3) | (4) | (5) | (6) | (7) |
| --- | --- | --- | --- | --- | --- | --- | --- |
| Global Coherence (1) |  | (-1.53, 1.53) | (-1.92, 2.06) | (-2.15, 2.29) | (-2.08, 2.15) | (-2.03, 1.98) | (-2.29, 2.17) |
| Individual Coherence (2) | (-1.53, 1.53) |  | (-1.57, 1.73) | (-1.96, 2.19) | (-1.80, 1.97) | (-1.79, 1.67) | (-2.09, 2.01) |
| Subjective Significance (3) | (-2.06, 1.92) | (-1.73, 1.57) |  | (-1.76, 1.67) | (-1.83, 1.86) | (-1.69, 1.49) | (-2.13, 2.00) |
| Objective Significance (4) | (-2.29, 2.15) | (-2.19, 1.96) | (-1.67, 1.76) |  | (-1.97, 2.06) | (-2.07, 1.89) | (-2.31, 2.03) |
| Meaning Direction (5) | (-2.15, 2.08) | (-1.97, 1.80) | (-1.86, 1.83) | (-2.06, 1.97) |  | (-1.68, 1.37) | (-2.14, 1.77) |
| Purpose Direction (6) | (-1.98, 2.03) | (-1.67, 1.79) | (-1.49, 1.69) | (-1.89, 2.07) | (-1.37, 1.68) |  | (-1.79, 1.41) |
| Goal Direction (7) | (-2.17, 2.29) | (-2.01, 2.09) | (-2.00, 2.13) | (-2.03, 2.31) | (-1.77, 2.14) | (-1.41, 1.79) |  |

Note: The average difference between quantiles is 3.80.

*Supplemental Table S6. QED Matrix for standardized CMM indicators*

| Item | (1) | (2) | (3) | (4) | (5) | (6) | (7) | (8) | (9) | (10) | (11) | (12) | (13) | (14) | (15) | (16) | (17) | (18) | (19) | (20) | (21) |
| --- | --- | --- | --- | --- | --- | --- | --- | --- | --- | --- | --- | --- | --- | --- | --- | --- | --- | --- | --- | --- | --- |
| CG1 (1) |  | (-1.26, 1.27) | (-2.10, 1.69) | (-1.66, 1.45) | (-1.99, 2.21) | (-2.04, 1.89) | (-2.25, 2.32) | (-2.51, 2.55) | (-2.30, 2.12) | (-2.21, 2.60) | (-2.41, 2.66) | (-2.52, 2.60) | (-1.96, 2.03) | (-2.30, 2.01) | (-2.16, 2.15) | (-2.01, 1.91) | (-2.25, 2.13) | (-2.51, 2.37) | (-2.08, 2.43) | (-2.32, 2.40) | (-2.41, 2.24) |
| CG2 (2) | (-1.27, 1.26) |  | (-2.13, 1.71) | (-1.69, 1.48) | (-2.01, 2.22) | (-2.06, 1.89) | (-2.27, 2.33) | (-2.55, 2.02) | (-1.73, 2.13) | (-2.12, 2.62) | (-2.45, 2.14) | (-2.55, 2.63) | (-1.97, 1.98) | (-2.33, 2.03) | (-2.19, 2.16) | (-2.03, 1.91) | (-2.28, 2.14) | (-2.54, 2.37) | (-2.70, 2.45) | (-2.35, 2.54) | (-2.43, 2.29) |
| CG3 (3) | (-1.69, 2.10) | (-1.71, 2.13) |  | (-1.61, 1.81) | (-1.88, 1.91) | (-1.94, 2.12) | (-2.15, 2.61) | (-2.47, 2.27) | (-2.21, 2.37) | (-2.62, 2.33) | (-2.37, 2.39) | (-2.47, 2.34) | (-1.81, 2.27) | (-2.25, 2.28) | (-2.11, 2.45) | (-1.93, 2.16) | (-2.20, 2.38) | (-2.46, 2.54) | (-2.62, 2.11) | (-2.21, 2.20) | (-2.31, 2.47) |
| CI1 (4) | (-1.45, 1.66) | (-1.48, 1.69) | (-1.81, 1.61) |  | (-2.19, 2.00) | (-2.19, 1.68) | (-1.83, 2.11) | (-2.10, 1.86) | (-1.88, 1.91) | (-2.30, 2.28) | (-2.05, 2.46) | (-2.10, 2.46) | (-2.11, 1.82) | (-2.45, 1.83) | (-2.35, 1.96) | (-2.17, 1.70) | (-1.84, 1.92) | (-2.09, 2.17) | (-2.25, 2.23) | (-2.40, 2.32) | (-2.56, 2.03) |
| CI2 (5) | (-2.21, 1.99) | (-2.22, 2.01) | (-1.91, 1.88) | (-2.00, 2.19) |  | (-1.92, 1.96) | (-2.02, 2.34) | (-2.23, 2.10) | (-2.16, 2.24) | (-2.48, 2.14) | (-2.24, 2.22) | (-2.32, 2.17) | (-2.36, 2.10) | (-2.61, 2.11) | (-2.49, 2.27) | (-2.35, 2.54) | (-2.11, 2.26) | (-2.25, 2.37) | (-2.50, 2.60) | (-2.70, 2.62) | (-2.85, 2.30) |
| CI3 (6) | (-1.89, 2.04) | (-1.89, 2.06) | (-2.12, 1.94) | (-1.68, 2.19) | (-1.96, 1.92) |  | (-2.22, 2.49) | (-2.53, 2.14) | (-1.71, 2.29) | (-2.14, 2.22) | (-2.43, 2.27) | (-2.52, 2.24) | (-1.89, 2.15) | (-2.32, 2.16) | (-2.18, 2.32) | (-1.99, 2.03) | (-2.23, 2.31) | (-2.53, 2.41) | (-2.68, 1.99) | (-2.30, 2.11) | (-2.38, 2.36) |
| SS1 (7) | (-2.32, 2.25) | (-2.33, 2.27) | (-2.61, 2.15) | (-2.11, 1.83) | (-2.34, 2.02) | (-2.49, 2.22) |  | (-2.32, 1.69) | (-1.52, 1.83) | (-2.49, 2.36) | (-2.24, 1.81) | (-2.33, 1.77) | (-2.36, 2.36) | (-2.13, 2.29) | (-2.06, 2.00) | (-1.80, 1.58) | (-2.08, 1.89) | (-2.34, 2.03) | (-2.50, 2.18) | (-2.10, 2.24) | (-2.19, 1.90) |
| SS2 (8) | (-2.55, 2.51) | (-2.02, 2.55) | (-2.27, 2.47) | (-1.86, 2.10) | (-2.10, 2.23) | (-2.14, 2.53) | (-1.69, 2.32) |  | (-1.79, 2.14) | (-2.20, 2.08) | (-1.90, 2.12) | (-2.03, 2.07) | (-2.03, 2.01) | (-2.38, 2.08) | (-2.25, 2.18) | (-2.12, 1.89) | (-2.36, 2.16) | (-2.57, 2.27) | (-2.81, 2.50) | (-2.43, 2.47) | (-2.50, 2.22) |
| SS3 (9) | (-2.12, 2.30) | (-2.13, 1.73) | (-2.37, 2.21) | (-1.91, 1.88) | (-2.24, 2.16) | (-2.29, 1.71) | (-1.83, 1.52) | (-2.14, 1.79) |  | (-2.35, 1.98) | (-2.04, 1.91) | (-2.15, 1.91) | (-2.16, 1.79) | (-1.93, 1.89) | (-2.36, 1.97) | (-2.24, 1.70) | (-1.88, 1.95) | (-2.14, 2.06) | (-2.29, 2.25) | (-2.47, 2.24) | (-2.02, 2.03) |
| SO1 (10) | (-2.60, 2.21) | (-2.62, 2.12) | (-2.33, 2.62) | (-2.28, 2.30) | (-2.14, 2.48) | (-2.22, 2.14) | (-2.36, 2.49) | (-2.08, 2.20) | (-1.98, 2.35) |  | (-1.90, 2.33) | (-2.01, 2.28) | (-2.09, 2.21) | (-2.25, 2.22) | (-2.26, 2.38) | (-2.19, 2.09) | (-2.37, 2.37) | (-2.72, 2.49) | (-2.23, 2.12) | (-2.43, 2.03) | (-2.58, 2.41) |
| SO2 (11) | (-2.66, 2.41) | (-2.14, 2.45) | (-2.39, 2.37) | (-2.46, 2.05) | (-2.22, 2.24) | (-2.27, 2.43) | (-1.81, 2.24) | (-2.12, 1.90) | (-1.91, 2.04) | (-2.33, 1.90) |  | (-2.13, 1.97) | (-2.14, 1.90) | (-2.50, 2.51) | (-2.37, 2.14) | (-2.24, 2.45) | (-2.49, 2.10) | (-2.20, 2.17) | (-2.28, 2.40) | (-2.55, 2.38) | (-2.63, 2.11) |
| SO3 (12) | (-2.60, 2.52) | (-2.63, 2.55) | (-2.34, 2.47) | (-2.46, 2.10) | (-2.17, 2.32) | (-2.24, 2.52) | (-1.77, 2.33) | (-2.07, 2.03) | (-1.91, 2.15) | (-2.28, 2.01) | (-1.97, 2.13) |  | (-2.09, 2.03) | (-2.45, 2.11) | (-2.32, 2.18) | (-2.19, 1.91) | (-2.44, 2.17) | (-2.13, 2.27) | (-2.25, 1.90) | (-2.50, 1.98) | (-2.59, 2.21) |
| DM1 (13) | (-2.03, 1.96) | (-1.98, 1.97) | (-2.27, 1.81) | (-1.82, 2.11) | (-2.10, 2.36) | (-2.15, 1.89) | (-2.36, 2.36) | (-2.01, 2.03) | (-1.79, 2.16) | (-2.21, 2.09) | (-1.90, 2.14) | (-2.03, 2.09) |  | (-1.78, 1.58) | (-2.25, 1.65) | (-2.12, 1.90) | (-1.78, 2.14) | (-2.08, 2.37) | (-2.16, 1.88) | (-2.43, 2.42) | (-2.51, 2.23) |
| DM2 (14) | (-2.01, 2.30) | (-2.03, 2.33) | (-2.28, 2.25) | (-1.83, 2.45) | (-2.11, 2.61) | (-2.16, 2.32) | (-2.29, 2.13) | (-2.08, 2.38) | (-1.89, 1.93) | (-2.22, 2.25) | (-2.51, 2.50) | (-2.11, 2.45) | (-1.58, 1.78) |  | (-1.65, 1.35) | (-2.13, 1.60) | (-1.78, 1.94) | (-2.16, 2.21) | (-2.16, 2.22) | (-2.29, 2.04) | (-2.45, 2.07) |
| DM3 (15) | (-2.15, 2.16) | (-2.16, 2.19) | (-2.45, 2.11) | (-1.96, 2.35) | (-2.27, 2.49) | (-2.32, 2.18) | (-2.00, 2.06) | (-2.18, 2.25) | (-1.97, 2.36) | (-2.38, 2.26) | (-2.14, 2.37) | (-2.18, 2.32) | (-1.65, 2.25) | (-1.35, 1.65) |  | (-1.69, 1.05) | (-1.91, 1.80) | (-2.17, 1.91) | (-2.27, 2.09) | (-2.47, 1.92) | (-2.62, 2.00) |
| DP1 (16) | (-1.91, 2.01) | (-1.91, 2.03) | (-2.16, 1.93) | (-1.70, 2.17) | (-2.54, 2.35) | (-2.03, 1.99) | (-1.58, 1.80) | (-1.89, 2.12) | (-1.70, 2.24) | (-2.09, 2.19) | (-2.45, 2.24) | (-1.91, 2.19) | (-1.90, 2.12) | (-1.60, 2.13) | (-1.05, 1.69) |  | (-1.55, 1.65) | (-1.88, 1.72) | (-2.04, 1.95) | (-2.24, 1.93) | (-2.39, 2.31) |
| DP2 (17) | (-2.13, 2.25) | (-2.14, 2.28) | (-2.38, 2.20) | (-1.92, 1.84) | (-2.26, 2.11) | (-2.31, 2.23) | (-1.89, 2.08) | (-2.16, 2.36) | (-1.95, 1.88) | (-2.37, 2.37) | (-2.10, 2.49) | (-2.17, 2.44) | (-2.14, 1.78) | (-1.94, 1.78) | (-1.80, 1.91) | (-1.65, 1.55) |  | (-2.15, 2.00) | (-2.28, 1.64) | (-2.48, 1.77) | (-2.05, 1.94) |
| DP3 (18) | (-2.37, 2.51) | (-2.37, 2.54) | (-2.54, 2.46) | (-2.17, 2.09) | (-2.37, 2.25) | (-2.41, 2.53) | (-2.03, 2.34) | (-2.27, 2.57) | (-2.06, 2.14) | (-2.49, 2.72) | (-2.17, 2.20) | (-2.27, 2.13) | (-2.37, 2.08) | (-2.21, 2.16) | (-1.91, 2.17) | (-1.72, 1.88) | (-2.00, 2.15) |  | (-1.76, 1.83) | (-1.96, 1.80) | (-2.18, 2.13) |
| DG1 (19) | (-2.43, 2.08) | (-2.45, 2.70) | (-2.11, 2.62) | (-2.23, 2.25) | (-2.60, 2.50) | (-1.99, 2.68) | (-2.18, 2.50) | (-2.50, 2.81) | (-2.25, 2.29) | (-2.12, 2.23) | (-2.40, 2.28) | (-1.90, 2.25) | (-1.88, 2.16) | (-2.22, 2.16) | (-2.09, 2.27) | (-1.95, 2.04) | (-1.64, 2.28) | (-1.83, 1.76) |  | (-2.19, 1.29) | (-2.20, 1.69) |
| DG2 (20) | (-2.40, 2.32) | (-2.54, 2.35) | (-2.20, 2.21) | (-2.32, 2.40) | (-2.62, 2.70) | (-2.11, 2.30) | (-2.24, 2.10) | (-2.47, 2.43) | (-2.24, 2.47) | (-2.03, 2.43) | (-2.38, 2.55) | (-1.98, 2.50) | (-2.42, 2.43) | (-2.04, 2.29) | (-1.92, 2.47) | (-1.93, 2.24) | (-1.77, 2.48) | (-1.80, 1.96) | (-1.29, 2.19) |  | (-1.71, 1.89) |
| DG3 (21) | (-2.24, 2.41) | (-2.29, 2.43) | (-2.47, 2.31) | (-2.03, 2.56) | (-2.30, 2.85) | (-2.36, 2.38) | (-1.90, 2.19) | (-2.22, 2.50) | (-2.03, 2.02) | (-2.41, 2.58) | (-2.11, 2.63) | (-2.21, 2.59) | (-2.23, 2.51) | (-2.07, 2.45) | (-2.00, 2.62) | (-2.31, 2.39) | (-1.94, 2.05) | (-2.13, 2.18) | (-1.69, 2.20) | (-1.89, 1.71) |  |

Note. The average difference between quantiles is 4.32.

Standardized QED proportions matrices for domains, subdomains and indicators are presented in the Supplementary Tables S7-S9.

*Supplemental Table S7. QED for proportion metrics for standardized CMM domain scores*

| Item | (1) | (2) | (3) |
| --- | --- | --- | --- |
| Coherence (1) |  | **6.5%** (3.7%,2.8%) | **6.0%** (2.9%,3.2%) |
| Significance (2) | **6.5%** (2.8%,3.7%) |  | **4.3%** (1.7%,2.6%) |
| Direction (3) | **6.0%** (3.2%,2.9%) | **4.3%** (2.6%,1.7%) |  |

*Supplemental Table S8. QED for proportion metrics for standardized CMM subdomain scores*

| Item | (1) | | (2) | | (3) | | (4) | | (5) | | (6) | | (7) | |  |
| --- | --- | --- | --- | --- | --- | --- | --- | --- | --- | --- | --- | --- | --- | --- | --- |
| Global Coherence (1) | |  | | **3.6%** (1.9%,1.7%) | | **8.8%** (4.4%,4.5%) | | **12.8%** (6.4%,6.4%) | | **9.0%** (5.0%,4.1%) | | **8.9%** (3.9%,4.9%) | | **14.0%** (6.0%,8.0%) | |
| Individual Coherence (2) | | **3.6%** (1.7%,1.9%) | |  | | **5.4%** (3.1%,2.3%) | | **9.6%** (4.2%,5.3%) | | **7.9%** (4.2%,3.7%) | | **5.8%** (3.1%,2.6%) | | **10.3%** (4.6%,5.7%) | |
| Subjective Significance (3) | | **8.8%** (4.5%,4.4%) | | **5.4%** (2.3%,3.1%) | |  | | **5.7%** (2.6%,3.0%) | | **7.7%** (4.0%,3.7%) | | **5.7%** (2.3%,3.3%) | | **9.6%** (4.3%,5.3%) | |
| Objective Significance (4) | | **12.8%** (6.4%,6.4%) | | **9.6%** (5.3%,4.2%) | | **5.7%** (3.0%,2.6%) | |  | | **8.9%** (4.8%,4.0%) | | **8.5%** (4.1%,4.4%) | | **11.0%** (4.5%,6.5%) | |
| Meaning Direction (5) | | **9.0%** (4.1%,5.0%) | | **7.9%** (3.7%,4.2%) | | **7.7%** (3.7%,4.0%) | | **8.9%** (4.0%,4.8%) | |  | | **3.8%** (1.2%,2.7%) | | **8.9%** (3.1%,5.8%) | |
| Purpose Direction (6) | | **8.9%** (4.9%,3.9%) | | **5.8%** (2.6%,3.1%) | | **5.7%** (3.3%,2.3%) | | **8.5%** (4.4%,4.1%) | | **3.8%** (2.7%,1.2%) | |  | | **4.6%** (1.8%,2.7%) | |
| Goal Direction (7) | | **14.0%** (8.0%,6.0%) | | **10.3%** (5.7%,4.6%) | | **9.6%** (5.3%,4.3%) | | **11.0%** (6.5%,4.5%) | | **8.9%** (5.8%,3.1%) | | **4.6%** (2.7%,1.8%) | |  | |

*Supplemental Table S9. QED for proportion metrics for standardized CMM item scores*

| Item | (1) | (2) | (3) | (4) | (5) | (6) | (7) | (8) | (9) | (10) | (11) | (12) | (13) | (14) | (15) | (16) | (17) | (18) | (19) | (20) | (21) |
| --- | --- | --- | --- | --- | --- | --- | --- | --- | --- | --- | --- | --- | --- | --- | --- | --- | --- | --- | --- | --- | --- |
| CG1 (1) |  | **3.2%** (1.3%,1.9%) | **6.5%** (2.9%,3.5%) | **7.9%** (1.9%,6.0%) | **13.0%** (5.0%,8.0%) | **11.0%** (5.1%,5.8%) | **10.9%** (6.2%,4.7%) | **15.2%** (6.5%,8.6%) | **12.0%** (3.8%,8.2%) | **16.5%** (8.2%,8.3%) | **17.0%** (6.4%,10.6%) | **17.1%** (7.9%,9.2%) | **11.4%** (5.4%,6.0%) | **16.4%** (6.1%,10.3%) | **10.1%** (4.5%,5.6%) | **12.8%** (6.2%,6.6%) | **7.3%** (3.6%,3.7%) | **17.3%** (6.0%,11.3%) | **15.8%** (8.5%,7.3%) | **16.0%** (8.2%,7.8%) | **19.5%** (6.3%,13.2%) |
| CG2 (2) | **3.2%** (1.9%,1.3%) |  | **6.1%** (2.7%,3.4%) | **8.3%** (2.4%,5.9%) | **13.2%** (5.1%,8.0%) | **11.3%** (5.2%,6.0%) | **12.2%** (7.6%,4.6%) | **14.3%** (6.0%,8.2%) | **12.0%** (3.8%,8.2%) | **15.8%** (8.0%,7.8%) | **16.5%** (6.6%,9.9%) | **16.5%** (7.8%,8.7%) | **10.9%** (5.1%,5.8%) | **15.0%** (5.8%,9.3%) | **9.6%** (4.4%,5.1%) | **12.3%** (6.1%,6.2%) | **7.7%** (3.3%,4.3%) | **17.2%** (6.2%,10.9%) | **16.9%** (9.2%,7.7%) | **16.1%** (8.6%,7.5%) | **19.3%** (6.4%,12.9%) |
| CG3 (3) | **6.5%** (3.5%,2.9%) | **6.1%** (3.4%,2.7%) |  | **7.7%** (5.9%,1.8%) | **13.8%** (6.6%,7.2%) | **7.9%** (3.3%,4.7%) | **12.3%** (7.7%,4.6%) | **14.1%** (4.7%,9.4%) | **12.2%** (8.8%,3.4%) | **14.2%** (5.7%,8.6%) | **22.9%** (11.9%,11.1%) | **19.2%** (9.5%,9.7%) | **11.5%** (4.5%,7.0%) | **12.9%** (6.8%,6.1%) | **15.4%** (9.3%,6.1%) | **11.5%** (4.6%,6.9%) | **12.0%** (8.1%,3.9%) | **19.5%** (9.2%,10.3%) | **15.3%** (6.8%,8.6%) | **13.0%** (6.1%,6.8%) | **15.4%** (9.6%,5.8%) |
| CI1 (4) | **7.9%** (6.0%,1.9%) | **8.3%** (5.9%,2.4%) | **7.7%** (1.8%,5.9%) |  | **9.9%** (4.9%,5.0%) | **7.2%** (3.1%,4.1%) | **10.2%** (3.9%,6.3%) | **12.2%** (7.0%,5.2%) | **7.2%** (3.4%,3.8%) | **16.4%** (8.5%,7.8%) | **13.6%** (7.6%,6.0%) | **14.4%** (8.8%,5.6%) | **10.6%** (7.0%,3.6%) | **13.2%** (6.8%,6.4%) | **12.6%** (4.8%,7.8%) | **7.6%** (4.4%,3.2%) | **7.5%** (3.0%,4.5%) | **12.8%** (5.9%,6.9%) | **9.1%** (3.6%,5.4%) | **15.1%** (5.1%,10.0%) | **15.1%** (6.4%,8.7%) |
| CI2 (5) | **13.0%** (8.0%,5.0%) | **13.2%** (8.0%,5.1%) | **13.8%** (7.2%,6.6%) | **9.9%** (5.0%,4.9%) |  | **10.6%** (5.9%,4.7%) | **14.8%** (9.0%,5.8%) | **11.5%** (5.9%,5.6%) | **11.2%** (5.4%,5.7%) | **16.7%** (6.8%,9.9%) | **10.9%** (5.3%,5.6%) | **12.2%** (6.8%,5.4%) | **13.4%** (7.5%,5.9%) | **16.5%** (8.2%,8.4%) | **16.7%** (8.9%,7.8%) | **14.3%** (7.7%,6.6%) | **12.1%** (5.7%,6.4%) | **10.8%** (4.9%,5.9%) | **16.5%** (9.4%,7.1%) | **16.8%** (7.8%,9.1%) | **14.8%** (6.9%,7.8%) |
| CI3 (6) | **11.0%** (5.8%,5.1%) | **11.3%** (6.0%,5.2%) | **7.9%** (4.7%,3.3%) | **7.2%** (4.1%,3.1%) | **10.6%** (4.7%,5.9%) |  | **10.6%** (7.4%,3.2%) | **12.7%** (5.2%,7.5%) | **9.5%** (4.3%,5.2%) | **12.7%** (6.0%,6.8%) | **14.4%** (5.3%,9.2%) | **14.3%** (6.3%,8.0%) | **11.9%** (6.0%,6.0%) | **10.5%** (5.3%,5.3%) | **14.0%** (9.2%,4.7%) | **10.2%** (5.1%,5.1%) | **8.7%** (5.7%,3.0%) | **17.4%** (8.8%,8.6%) | **13.8%** (7.2%,6.6%) | **12.1%** (6.8%,5.4%) | **21.6%** (10.4%,11.1%) |
| SS1 (7) | **10.9%** (4.7%,6.2%) | **12.2%** (4.6%,7.6%) | **12.3%** (4.6%,7.7%) | **10.2%** (6.3%,3.9%) | **14.8%** (5.8%,9.0%) | **10.6%** (3.2%,7.4%) |  | **12.2%** (4.8%,7.4%) | **6.4%** (4.2%,2.2%) | **14.4%** (6.2%,8.2%) | **8.6%** (5.7%,3.0%) | **15.5%** (7.9%,7.7%) | **18.0%** (9.3%,8.6%) | **13.5%** (7.8%,5.7%) | **13.3%** (6.9%,6.3%) | **8.8%** (2.4%,6.4%) | **10.3%** (5.8%,4.5%) | **13.3%** (5.5%,7.8%) | **13.0%** (4.2%,8.8%) | **12.8%** (4.8%,8.0%) | **14.2%** (7.9%,6.3%) |
| SS2 (8) | **15.2%** (8.6%,6.5%) | **14.3%** (8.2%,6.0%) | **14.1%** (9.4%,4.7%) | **12.2%** (5.2%,7.0%) | **11.5%** (5.6%,5.9%) | **12.7%** (7.5%,5.2%) | **12.2%** (7.4%,4.8%) |  | **7.6%** (3.5%,4.1%) | **10.2%** (5.5%,4.7%) | **10.7%** (4.9%,5.8%) | **11.1%** (5.8%,5.3%) | **11.6%** (6.4%,5.2%) | **14.1%** (7.0%,7.1%) | **9.9%** (5.1%,4.8%) | **10.2%** (6.0%,4.2%) | **12.7%** (4.9%,7.8%) | **12.9%** (5.4%,7.4%) | **14.3%** (8.7%,5.5%) | **18.2%** (8.5%,9.7%) | **14.9%** (5.4%,9.4%) |
| SS3 (9) | **12.0%** (8.2%,3.8%) | **12.0%** (8.2%,3.8%) | **12.2%** (3.4%,8.8%) | **7.2%** (3.8%,3.4%) | **11.2%** (5.7%,5.4%) | **9.5%** (5.2%,4.3%) | **6.4%** (2.2%,4.2%) | **7.6%** (4.1%,3.5%) |  | **12.1%** (6.1%,6.0%) | **9.6%** (5.4%,4.2%) | **10.3%** (6.7%,3.6%) | **9.0%** (6.0%,3.0%) | **11.8%** (5.8%,6.0%) | **10.7%** (4.0%,6.8%) | **6.5%** (3.8%,2.6%) | **8.4%** (3.3%,5.1%) | **10.4%** (5.2%,5.3%) | **7.5%** (3.0%,4.5%) | **15.1%** (4.8%,10.3%) | **13.2%** (5.5%,7.7%) |
| SO1 (10) | **16.5%** (8.3%,8.2%) | **15.8%** (7.8%,8.0%) | **14.2%** (8.6%,5.7%) | **16.4%** (7.8%,8.5%) | **16.7%** (9.9%,6.8%) | **12.7%** (6.8%,6.0%) | **14.4%** (8.2%,6.2%) | **10.2%** (4.7%,5.5%) | **12.1%** (6.0%,6.1%) |  | **11.0%** (6.3%,4.7%) | **7.2%** (3.6%,3.5%) | **11.5%** (5.5%,5.9%) | **12.2%** (6.5%,5.7%) | **10.6%** (6.4%,4.3%) | **10.0%** (5.7%,4.3%) | **12.4%** (6.5%,5.9%) | **17.3%** (10.1%,7.2%) | **13.5%** (7.6%,5.9%) | **18.0%** (6.5%,11.4%) | **19.6%** (9.8%,9.8%) |
| SO2 (11) | **17.0%** (10.6%,6.4%) | **16.5%** (9.9%,6.6%) | **22.9%** (11.1%,11.9%) | **13.6%** (6.0%,7.6%) | **10.9%** (5.6%,5.3%) | **14.4%** (9.2%,5.3%) | **8.6%** (3.0%,5.7%) | **10.7%** (5.8%,4.9%) | **9.6%** (4.2%,5.4%) | **11.0%** (4.7%,6.3%) |  | **6.4%** (3.4%,3.1%) | **12.4%** (7.5%,4.9%) | **16.2%** (7.6%,8.6%) | **15.0%** (5.8%,9.2%) | **11.1%** (6.5%,4.6%) | **13.8%** (5.4%,8.4%) | **11.7%** (4.9%,6.8%) | **13.1%** (8.3%,4.8%) | **17.5%** (6.4%,11.2%) | **15.1%** (5.7%,9.5%) |
| SO3 (12) | **17.1%** (9.2%,7.9%) | **16.5%** (8.7%,7.8%) | **19.2%** (9.7%,9.5%) | **14.4%** (5.6%,8.8%) | **12.2%** (5.4%,6.8%) | **14.3%** (8.0%,6.3%) | **15.5%** (7.7%,7.9%) | **11.1%** (5.3%,5.8%) | **10.3%** (3.6%,6.7%) | **7.2%** (3.5%,3.6%) | **6.4%** (3.1%,3.4%) |  | **10.4%** (5.3%,5.0%) | **14.6%** (5.8%,8.8%) | **13.2%** (5.7%,7.4%) | **10.2%** (5.7%,4.5%) | **13.3%** (4.3%,8.9%) | **12.1%** (4.7%,7.4%) | **12.3%** (7.0%,5.3%) | **19.4%** (7.2%,12.2%) | **14.8%** (4.8%,10.0%) |
| DM1 (13) | **11.4%** (6.0%,5.4%) | **10.9%** (5.8%,5.1%) | **11.5%** (7.0%,4.5%) | **10.6%** (3.6%,7.0%) | **13.4%** (5.9%,7.5%) | **11.9%** (6.0%,6.0%) | **18.0%** (8.6%,9.3%) | **11.6%** (5.2%,6.4%) | **9.0%** (3.0%,6.0%) | **11.5%** (5.9%,5.5%) | **12.4%** (4.9%,7.5%) | **10.4%** (5.0%,5.3%) |  | **7.5%** (2.3%,5.2%) | **5.9%** (2.6%,3.3%) | **8.6%** (4.0%,4.6%) | **9.7%** (2.5%,7.2%) | **13.3%** (5.3%,8.1%) | **12.3%** (7.1%,5.1%) | **18.2%** (7.4%,10.8%) | **16.0%** (5.1%,10.9%) |
| DM2 (14) | **16.4%** (10.3%,6.1%) | **15.0%** (9.3%,5.8%) | **12.9%** (6.1%,6.8%) | **13.2%** (6.4%,6.8%) | **16.5%** (8.4%,8.2%) | **10.5%** (5.3%,5.3%) | **13.5%** (5.7%,7.8%) | **14.1%** (7.1%,7.0%) | **11.8%** (6.0%,5.8%) | **12.2%** (5.7%,6.5%) | **16.2%** (8.6%,7.6%) | **14.6%** (8.8%,5.8%) | **7.5%** (5.2%,2.3%) |  | **4.8%** (1.0%,3.8%) | **6.2%** (2.4%,3.7%) | **9.7%** (4.0%,5.7%) | **13.7%** (5.7%,8.0%) | **10.0%** (4.6%,5.4%) | **14.1%** (4.3%,9.8%) | **16.2%** (5.8%,10.4%) |
| DM3 (15) | **10.1%** (5.6%,4.5%) | **9.6%** (5.1%,4.4%) | **15.4%** (6.1%,9.3%) | **12.6%** (7.8%,4.8%) | **16.7%** (7.8%,8.9%) | **14.0%** (4.7%,9.2%) | **13.3%** (6.3%,6.9%) | **9.9%** (4.8%,5.1%) | **10.7%** (6.8%,4.0%) | **10.6%** (4.3%,6.4%) | **15.0%** (9.2%,5.8%) | **13.2%** (7.4%,5.7%) | **5.9%** (3.3%,2.6%) | **4.8%** (3.8%,1.0%) |  | **3.8%** (0.8%,3.0%) | **8.1%** (4.4%,3.7%) | **10.2%** (4.5%,5.7%) | **12.1%** (3.3%,8.8%) | **11.4**% (3.1%,8.3%) | **14.0%** (6.4%,7.6%) |
| DP1 (16) | **12.8%** (6.6%,6.2%) | **12.3%** (6.2%,6.1%) | **11.5%** (6.9%,4.6%) | **7.6%** (3.2%,4.4%) | **14.3%** (6.6%,7.7%) | **10.2%** (5.1%,5.1%) | **8.8%** (6.4%,2.4%) | **10.2%** (4.2%,6.0%) | **6.5%** (2.6%,3.8%) | **10.0%** (4.3%,5.7%) | **11.1%** (4.6%,6.5%) | **10.2%** (4.5%,5.7%) | **8.6%** (4.6%,4.0%) | **6.2%** (3.7%,2.4%) | **3.8% (**3.0%,0.8%) |  | **3.1%** (1.9%,1.3%) | **10.4%** (4.0%,6.4%) | **8.3%** (4.3%,4.0%) | **7.6%** (3.4%,4.1%) | **14.8%** (6.2%,8.6%) |
| DP2 (17) | **7.3%** (3.7%,3.6%) | **7.7%** (4.3%,3.3%) | **12.0%** (3.9%,8.1%) | **7.5%** (4.5%,3.0%) | **12.1%** (6.4%,5.7%) | **8.7%** (3.0%,5.7%) | **10.3%** (4.5%,5.8%) | **12.7%** (7.8%,4.9%) | **8.4%** (5.1%,3.3%) | **12.4%** (5.9%,6.5%) | **13.8% (**8.4%,5.4%) | **13.3%** (8.9%,4.3%) | **9.7%** (7.2%,2.5%) | **9.7%** (5.7%,4.0%) | **8.1%** (3.7%,4.4%) | **3.1%** (1.3%,1.9%) |  | **8.8%** (4.1%,4.7%) | **7.6%** (2.3%,5.3%) | **12.1%** (3.4%,8.7%) | **12.7%** (5.0%,7.6%) |
| DP3 (18) | **17.3%** (11.3%,6.0%) | **17.2%** (10.9%,6.2%) | **19.5%** (10.3%,9.2%) | **12.8%** (6.9%,5.9%) | **10.8%** (5.9%,4.9%) | **17.4%** (8.6%,8.8%) | **13.3%** (7.8%,5.5%) | **12.9%** (7.4%,5.4%) | **10.4%** (5.3%,5.2%) | **17.3%** (7.2%,10.1%) | **11.7%** (6.8%,4.9%) | **12.1%** (7.4%,4.7%) | **13.3%** (8.1%,5.3%) | **13.7%** (8.0%,5.7%) | **10.2%** (5.7%,4.5%) | **10.4%** (6.4%,4.0%) | **8.8%** (4.7%,4.1%) |  | **9.7%** (5.2%,4.5%) | **9.9%** (4.8%,5.0%) | **7.7%** (3.4%,4.3%) |
| DG1 (19) | **15.8%** (7.3%,8.5%) | **16.9%** (7.7%,9.2%) | **15.3%** (8.6%,6.8%) | **9.1%** (5.4%,3.6%) | **16.5%** (7.1%,9.4%) | **13.8%** (6.6%,7.2%) | **13.0%** (8.8%,4.2%) | **14.3%** (5.5%,8.7%) | **7.5%** (4.5%,3.0%) | **13.5%** (5.9%,7.6%) | **13.1%** (4.8%,8.3%) | **12.3%** (5.3%,7.0%) | **12.3%** (5.1%,7.1%) | **10.0%** (5.4%,4.6%) | **12.1%** (8.8%,3.3%) | **8.3%** (4.0%,4.3%) | **7.6%** (5.3%,2.3%) | **9.7%** (4.5%,5.2%) |  | **4.7%** (1.3%,3.4%) | **11.3%** (4.3%,7.0%) |
| DG2 (20) | **16.0%** (7.8%,8.2%) | **16.1%** (7.5%,8.6%) | **13.0%** (6.8%,6.1%) | **15.1%** (10.0%,5.1%) | **16.8%** (9.1%,7.8%) | **12.1%** (5.4%,6.8%) | **12.8%** (8.0%,4.8%) | **18.2%** (9.7%,8.5%) | **15.1%** (10.3%,4.8%) | **18.0%** (11.4%,6.5%) | **17.5%** (11.2%,6.4%) | **19.4%** (12.2%,7.2%) | **18.2%** (10.8%,7.4%) | **14.1%** (9.8%,4.3%) | **11.4%** (8.3%,3.1%) | **7.6%** (4.1%,3.4%) | **12.1%** (8.7%,3.4%) | **9.9%** (5.0%,4.8%) | **4.7%** (3.4%,1.3%) |  | **8.3%** (5.2%,3.1%) |
| DG3 (21) | **19.5%** (13.2%,6.3%) | **19.3%** (12.9%,6.4%) | **15.4%** (5.8%,9.6%) | **15.1%** (8.7%,6.4%) | **14.8%** (7.8%,6.9%) | **21.6%** (11.1%,10.4%) | **14.2%** (6.3%,7.9%) | **14.9%** (9.4%,5.4%) | **13.2%** (7.7%,5.5%) | **19.6%** (9.8%,9.8%) | **15.1%** (9.5%,5.7%) | **14.8%** (10.0%,4.8%) | **16.0%** (10.9%,5.1%) | **16.2%** (10.4%,5.8%) | **14.0%** (7.6%,6.4%) | **14.8%** (8.6%,6.2%) | **12.7%** (7.6%,5.0%) | **7.7%** (4.3%,3.4%) | **11.3%** (7.0%,4.3%) | **8.3%** (3.1%,5.2%) |  |

**Example R Code to Implement QED Analyses**.

See

library(tidyverse)

library(flextable)

library(data.table)

library(lavaan)

## Wrapper functions

compute_qed <- function(data, quant=0.025, mylabels=NULL, stand=FALSE){

ITEMS.i <- colnames(data)

if(is.null(mylabels)) mylabels <- ITEMS.i

if(stand){

data = data %>%

mutate(

across(everything(), \(x) scale(x) )

)

}

out.dat <- data.frame(matrix(ncol=length(ITEMS.i)+1, nrow=length(ITEMS.i)))

colnames(out.dat) <- c("Item", paste0("(",1:length(ITEMS.i),")"))

out.dat$Item <- mylabels

i <- j <- 1

diff.dat <- matrix(nrow=nrow(out.dat), ncol=ncol(out.dat)-1)

for(i in 1:length(ITEMS.i)){

for(j in 1:length(ITEMS.i)){

if(i == j){

next

} else{

sub_dat <- data %>%

mutate(

diff = .data[[ITEMS.i[i]]] - .data[[ITEMS.i[j]]]

) %>% summarise(

q1 = quantile(diff, quant, na.rm=T),

q2 = quantile(diff, 1-quant, na.rm=T)

)

diff.dat[i,j] <- sub_dat$q2[1] - sub_dat$q1[1]

out.dat[i,j+1] <- paste0("(", .round(sub_dat$q1[1]) ,", ", .round(sub_dat$q2[1]), ")")

}

}

}

out.dat

}

qed_avg_diff <- function(data, quant=0.025, mylabels=NULL, stand=FALSE){

ITEMS.i <- colnames(data)

if(is.null(mylabels)) mylabels <- ITEMS.i

if(stand){

data = data %>%

mutate(

across(everything(), \(x) scale(x) )

)

}

out.dat <- data.frame(matrix(ncol=length(ITEMS.i)+1, nrow=length(ITEMS.i)))

colnames(out.dat) <- c("Item", paste0("(",1:length(ITEMS.i),")"))

out.dat$Item <- mylabels

i <- j <- 1

diff.dat <- matrix(nrow=nrow(out.dat), ncol=ncol(out.dat)-1)

for(i in 1:length(ITEMS.i)){

for(j in 1:length(ITEMS.i)){

if(i == j){

next

} else{

sub_dat <- data %>%

mutate(

diff = .data[[ITEMS.i[i]]] - .data[[ITEMS.i[j]]]

) %>% summarise(

q1 = quantile(diff, quant, na.rm=T),

q2 = quantile(diff, 1-quant, na.rm=T)

)

diff.dat[i,j] <- sub_dat$q2[1] - sub_dat$q1[1]

}

}

}

mean(abs(diff.dat[lower.tri(diff.dat)]), na.rm=TRUE)

}

compute_qed_proportion_metric <- function(data, cutoff=NULL, mylabels=NULL, stand=FALSE, directional = TRUE, concat=FALSE,...){

ITEMS.i <- colnames(data)

if(is.null(mylabels)) mylabels <- ITEMS.i

if(stand){

data = data %>%

mutate(

across(everything(), \(x) scale(x) )

)

if(is.null(cutoff)) cutoff=1.65

}

if(is.null(cutoff)) cutoff = 2

out.dat <- data.frame(matrix(ncol=length(ITEMS.i)+1, nrow=length(ITEMS.i)))

colnames(out.dat) <- c("Item", paste0("(",1:length(ITEMS.i),")"))

out.dat$Item <- mylabels

i <- j <- 1

for(i in 1:length(ITEMS.i)){

for(j in 1:length(ITEMS.i)){

if(i == j){

next

} else{

sub_dat <- data %>%

mutate(

diff = .data[[ITEMS.i[i]]] - .data[[ITEMS.i[j]]]

)

if(directional){

sub_dat <- sub_dat %>%

summarise(

q2 = mean( diff >= cutoff, na.rm=T)

)

} else {

sub_dat <- sub_dat %>%

summarise(

q2 = mean( abs(diff) >= cutoff, na.rm=T)

)

}

out.dat[i,j+1] <- sub_dat$q2[1]

}

}

}

if(concat == TRUE){

out.dat <- data.frame(matrix(ncol=length(ITEMS.i)+1, nrow=length(ITEMS.i)))

colnames(out.dat) <- c("Item", paste0("(",1:length(ITEMS.i),")"))

out.dat$Item <- mylabels

i <- j <- 1

for(i in 1:length(ITEMS.i)){

for(j in 1:length(ITEMS.i)){

if(i == j){

next

} else{

sub_dat <- data %>%

mutate(

diff = .data[[ITEMS.i[i]]] - .data[[ITEMS.i[j]]]

) %>%

summarise(

p_ij = mean( diff >= cutoff, na.rm=T),

p_ji = mean( diff <= -cutoff, na.rm=T)

)

out.dat[i,j+1] <- paste0("(",.round(round(sub_dat$p_ij[1],3)*100,1), "%,", .round(round(sub_dat$p_ji[1],3)*100,1), "%",")")

}

}

}

}

out.dat

}

## ========================================================================== ##

## ========================================================================== ##

# (LOAD DATA - NEED TO REPLACE WITH YOUR OWN DATA)

temp.dat <- readr::read_rds("data/hrs_data_cleaned.rds")

# a labeling vector

BETTER.LABELS <-

c(

"Life is close to ideal (1)",

"Life conditions are excellent (2)",

"Satisfied with life (3)",

"Have important things in life (4)",

"Change none if lived life over (5)"

)

## ========================================================================== ##

## ========================================================================== ##

## Observed correlations

# "SWL observed correlations"

cor.out <- as.data.frame(matrix(ncol=length(BETTER.LABELS)+1,nrow=length(BETTER.LABELS)))

colnames(cor.out) <- c("Item", paste0("(",1:length(BETTER.LABELS),")"))

cor.out$Item <- BETTER.LABELS

Cij <- cor(temp.dat, use="pairwise.complete.obs")

cor.out[,2:(length(BETTER.LABELS)+1)] <- Cij

diag(cor.out[,2:(length(BETTER.LABELS)+1)]) <- NA

cor.out

temp.dat %>%

summarise(

across(everything(), \(x) mean(x, na.rm=TRUE))

)

temp.dat %>%

summarise(

across(everything(), \(x) sd(x, na.rm=TRUE))

)

## QED Unstandardized

# "SWL QED with unstandardized indicators"

out.dat <- compute_qed(

data = temp.dat,

quant=0.025,

mylabels=BETTER.LABELS

)

# read as (row - column)

out.dat

cat("\nAverage difference between quantiles:\t",round(qed_avg_diff(temp.dat, 0.025)) )

## QED Standardized

# "SWL QED with standardized indicators"

out.dat <- compute_qed(

data = temp.dat,

quant=0.025, stand=TRUE,

mylabels=BETTER.LABELS

)

# read as (row - column)

out.dat

cat("\nAverage difference between quantiles:\t",round(qed_avg_diff(temp.dat, 0.025, stand=TRUE)) )

## Proportion of Sample with 3+ pt differences

# "SWL proportion of sample with 3+ pt differences"

compute_qed_proportion_metric(

data = temp.dat,

cutoff=3,

mylabels=BETTER.LABELS,

concat=TRUE

)

# SWL proportion of sample responding 1.65 SD STANDARDIZED

compute_qed_proportion_metric(

data = temp.dat,

cutoff=1.65, stand=TRUE,

mylabels=BETTER.LABELS,

concat=TRUE

)

## Factor Analysis Benchmarking matrix

ITEMS.i <- colnames(temp.dat)

mod <- "

swl =~ RLB002A + RLB002B + RLB002C + RLB002D + RLB002E

"

fit <- sem(mod, data=temp.dat, meanstructure=T)

# model implied difference distribution

lambda <- parameterEstimates(fit) %>% filter(op == "=~") %>% select(est)

lambda.std <- standardizedSolution(fit) %>% filter(op == "=~") %>% select(est.std)

EX <- parameterEstimates(fit) %>% filter(op == "~1", lhs!="swl") %>% select(est)

VX <- parameterEstimates(fit) %>% filter(op == "~~", lhs != "swl") %>% select(est)

Veta <- parameterEstimates(fit) %>% filter(op == "~~", lhs == "swl") %>% select(est)

Vcov <- fit@implied$cov[[1]]

# implied distribution

nV = nrow(EX)

out.dat = out.dat.quantiles = obs.diff = exp.diff = prop.diff = q1 = q2 = matrix(nrow=nV, ncol=nV)

for(i in 1:nV){

for(j in 1:nV){

if(i != j){

# part 1. observed difference between QED limits

sub_dat <- temp.dat %>%

mutate(

diff = .data[[ITEMS.i[i]]] - .data[[ITEMS.i[j]]]

) %>% summarise(

q1 = quantile(diff, 0.025, na.rm=T),

q2 = quantile(diff, 0.975, na.rm=T)

)

obs.diff[i,j] <- sub_dat$q2[1] - sub_dat$q1[1]

# part 2. expected difference between QED limits

q1[i,j] <- qnorm(

0.025,

mean = EX[i,1] - EX[j,1],

sd = sqrt(Vcov[i,i] + Vcov[j,j] - 2*Vcov[i,j])

)

q2[i,j] <- qnorm(

0.975,

mean = EX[i,1] - EX[j,1],

sd = sqrt(Vcov[i,i] + Vcov[j,j] - 2*Vcov[i,j])

)

exp.diff[i,j] <- q2[i,j] - q1[i,j]

# 3. proportion of the way that the observed difference between standardized limits is between expected difference and 5.54 (expected difference for two uncorrelated normal variables)

prop.diff[i,j] <- (obs.diff[i,j] - exp.diff[i,j])/(5.54 - exp.diff[i,j]) * 100

# combined into output matrix.

out.dat[i,j] = paste0("[",round(obs.diff[i,j],1), ",",round(exp.diff[i,j],1),", ", round(prop.diff[i,j],0) ,"%]")

out.dat.quantiles[i,j] <- paste0("(",round(q1[i,j],2), ", ", round(q2[i,j],2) ,")")

}

}

}

rownames(out.dat) <- BETTER.LABELS

colnames(out.dat) <- paste0("(",1:length(BETTER.LABELS),")")

out.dat <- as.data.frame(out.dat) %>%

rownames_to_column(var="Item")

out.dat$Loading.STD = lambda.std$est.std

out.dat

rownames(out.dat.quantiles) <- BETTER.LABELS

colnames(out.dat.quantiles) <- paste0("(",1:length(BETTER.LABELS),")")

out.dat.quantiles <- as.data.frame(out.dat.quantiles) %>%

rownames_to_column(var="Item")

out.dat.quantiles
